# Development and validation of a search filter to identify equity-focused studies: reducing the number needed to screen

**DOI:** 10.1186/s12874-018-0567-x

**Published:** 2018-10-12

**Authors:** Stephanie L Prady, Eleonora P Uphoff, Madeleine Power, Su Golder

**Affiliations:** Department of Health Sciences, University of York, York, YO10 5DD UK

**Keywords:** Search filters, hedges, MEDLINE, Embase, Information storage and retrieval, literature searching, equity, health inequalities, systematic review, social determinants of health

## Abstract

**Background:**

Health inequalities, worse health associated with social and economic disadvantage, are reported by a minority of research articles. Locating these studies when conducting an equity-focused systematic review is challenging due to a deficit in standardised terminology, indexing, and lack of validated search filters. Current reporting guidelines recommend not applying filters, meaning that increased resources are needed at the screening stage.

**Methods:**

We aimed to design and test search filters to locate studies that reported outcomes by a social determinant of health. We developed and expanded a ‘specific terms strategy’ using keywords and subject headings compiled from recent systematic reviews that applied an equity filter. A ‘non-specific strategy’ was compiled from phrases used to describe equity analyses that were reported in titles and abstracts, and related subject headings. Gold standard evaluation and validation sets were compiled. The filters were developed in MEDLINE, adapted for Embase and tested in both. We set a target of 0.90 sensitivity (95% CI; 0.84, 0.94) in retrieving 150 gold standard validation papers. We noted the reduction in the number needed to screen in a proposed equity-focused systematic review and the proportion of equity-focused reviews we assessed in the project that applied an equity filter to their search strategy.

**Results:**

The specific terms strategy filtered out 93-95% of all records, and retrieved a validation set of articles with a sensitivity of 0.84 in MEDLINE (0.77, 0.89), and 0.87 (0.81, 0.92) in Embase. When combined (Boolean ‘OR’) with the non-specific strategy sensitivity was 0.92 (0.86, 0.96) in MEDLINE (Embase 0.94; 0.89, 0.97). The number needed to screen was reduced by 77% by applying the specific terms strategy, and by 59.7% (MEDLINE) and 63.5% (Embase) by applying the combined strategy. Eighty-one per cent of systematic reviews filtered studies by equity.

**Conclusions:**

A combined approach of using specific and non-specific terms is recommended if systematic reviewers wish to filter studies for reporting outcomes by social determinants. Future research should concentrate on the indexing standardisation for equity studies and further development and testing of both specific and non-specific terms for accurate study retrieval.

**Electronic supplementary material:**

The online version of this article (10.1186/s12874-018-0567-x) contains supplementary material, which is available to authorized users.

## Background

Fewer than fifteen per cent of intervention studies report any outcome by a social determinant of health [1]. We label the notion of unfair and avoidable differences in health by socioeconomic group as ‘equity’ throughout this paper, noting that the terms health inequalities, inequities, and disparities are increasingly used interchangeably [2]. Locating this minority of studies within the literature is a challenge that is hampered by the absence of validated equity search filters [3]. The current Preferred Reporting Items for Systematic Reviews and Meta-Analyses equity extension guidance (PRISMA-E) recommends that systematic reviewers do not attempt to filter searches due to poor indexing of equity terms which would lead to relevant studies being missed [4]. It is unclear how many systematic reviewers follow this advice. An obvious disadvantage to the advised sensitive (non-filtered) approach to study identification is that increased resources are needed at the screening stage, which can place a considerable burden on the research team [5].

The motivation to design and test the performance of an equity filter arose from plans to conduct a multi-disciplinary systematic review. This review aimed to examine the effect of any medical, screening or health service intervention, or any social or policy intervention that reported the prevention or reduction in the burden of mental health inequalities in UK adults. Filters were applied for study design and participant type (adult, humans); but not for the type of intervention and outcome measures as these needed to be as broad as possible. Scoping searches revealed that upwards of fifty thousand records would be retrieved, too vast for the limited resource available to independently screen studies. A crude unevaluated filter for health inequalities was observed to reduce the number of records retrieved in MEDLINE and Embase tenfold. As a result, it was decided to attempt to develop a filter to identify equity-focused studies. The sensitivity of the filter, i.e. how many relevant records it retrieved, was of paramount interest.

We therefore aimed to build and test a search strategy to identify studies that reported health inequalities, and assess the sensitivity of the filter against a gold-standard set of equity-focused studies. Secondary aims were to (1) report the reduction in the number needed to screen as a result of adding the filter to a systematic review search strategy, and (2) report on the proportion of published equity-focused systematic reviews that used an equity filter.

## Methods

There are four stages of search filter development [6]:

1. Search term selection
2. Identification of a ‘gold standard’
3. Evaluation of the filter
4. Validation of the filter.

### Search term selection

We sought to evaluate two complementary sets of terms; one ‘specific’ set of terms relating to words used to describe the factors of interest (the social determinants of health) and a ‘non-specific’ set of terms relating to words used to describe the aims, methods or results of equity analyses.

#### Specific terms

The goal of this stage was to populate the categories of interest with search terms. We adapted a pragmatic approach of using terms reported in published search strategies [7] into an iterative strategy of identifying terms included in systematic reviews.

First, the search strategies of equity-focused systematic reviews published within the most recent year were examined. These reviews were identified via Embase and MEDLINE (OVID) on 3.10.2017 using the search terms:

(equit* or inequal* or inequit* or socioeconomic or socio$economic or dispar*).ti. AND (systematic adj review).ti., limited to English language, excluding conference abstracts, and published in 2017.

Data extracted by one author (SLP) included; the citation, whether an equity filter was applied, and whether an example strategy for the equity filter was made available. Terms in English relating to the social determinants of health used in the published example search strategy were extracted. If a strategy for more than one database was provided then terms for only one was extracted (in all cases this was MEDLINE). Terms relating to other exposures examined in the reviews were ignored, unless this was also a term of interest.

Inequality terms were classified into concepts and measures where possible and classified into PROGRESS categories by one author (SLP). ‘PROGRESS’ is an acronym for common situations and circumstances associated with health inequality that can be used by authors of equity-focused systematic reviews to categorise social determinants of health [4]. The acronym refers to Place of residence, Race /ethnicity, Occupation, Gender, Religion, Education, Socio-economic status and Social capital. In ‘Place of residence’ we included terms such as marital or cohabitation status and the size of the household, and included income under ‘Socio-economic status’. The three further categories, the –Plus in PROGRESS-Plus, refers to factors that may put individuals at disadvantage. These are listed as; (1) personal characteristics such as age or disability, (2) situations resulting from interdependent relationships and (3) time-limited disadvantage resulting from a temporary change in circumstance [8]. As a pragmatic step, all the -Plus groupings were dropped because they could encompass almost limitless situations and demographics. Terms that did not map to PROGRESS categories (and were not related to -Plus) were classified under an additional ‘Other’ category.

A second search was undertaken to identify further equity-focused reviews to provide terms for unpopulated or sparsely populated (≤3 studies contributing terms) PROGRESS categories. This search (carried out on 19.10.2017) was based on the strategy above but for reviews published in 2016.

A third set of category-specific searches were undertaken to identify key terms for categories that remained un- or sparsely populated. These searches aimed to identify any systematic reviews, not just those with an equity-focus, which included basic terms relating to the desired category in the title. The search was carried out in Embase and MEDLINE (OVID) on 19.10.2017 using the search terms:

([*PROGRESS keyword*]ti. AND (systematic adj review).ti., limited to English language, excluding conference abstracts, and published between 2012 and 2015.

In the event that very large numbers of reviews were retrieved from the targeted searches, reviews were randomly ordered using a list randomiser (https://www.random.org/lists/) and sequentially inspected until five reviews presenting the terms of interest were included.

The strategy was developed in MEDLINE. Included Medical Subject Headings (MeSH) terms were verified in the MeSH browser (meshb.nlm.nih.gov) and related tree structures inspected to determine if the term should be exploded i.e. all nested concepts were relevant, or not. When examining the tree structures, we noted related subject headings and added those that were missing from our included compilation. We examined each term for face validity and removed those that we did not consider to be a social determinant of health, and those that were too general. We adapted the strategy to be compatible with both American English and English spelling, and truncated terms where appropriate to capture both singular and plural endings and for similar stem terms.

Categorised terms were compiled into a search strategy for MEDLINE by SLP which was checked for errors by SG. The search was carried out in OVID MEDLINE by SLP without knowledge of which articles were in the gold standard.

#### Non-specific terms

We compiled terms and phrases reported in titles and abstracts, and MeSH descriptors, which were associated with the reporting of differential effects from papers included in the two evaluation sets (see below). Examples of these phrases were ‘…risk factors for…’, ‘…differed by…’, and ‘…significant among…’.

### Gold standard sample size

The overall aim of the study was to calculate the sensitivity of the filter against the gold standard set of articles [6]:

$$ \frac{No. of\ relevant\ records\ retrieved\  by\  the\ filter}{No. of\ records\ in\ the\ gold\ standard}\ \mathrm{x}\ 100 $$

We considered that the retrieval of 90% of relevant records would be a good result for this filter. Ninety-five per cent binomial confidence intervals around a sensitivity of 0.90 were 0.84 to 0.94 for a sample size of 150 gold standard papers.

### Identification of a ‘gold standard’ set of papers

We used similar methods to identify the gold standard papers for use in both the development and validation of the filter (internal standard). Identification of the gold standard papers was carried out independently by two reviewers (MP and EPU) blind to SLP who compiled the search terms, and who were themselves blinded to the compilation of the search terms. MP and EU are experienced researchers who have published on social epidemiology. Reliability was not formally assessed.

Three strategies were employed. First, the ‘relative recall’ method was used, identifying relevant studies from a range of previously published systematic reviews [5]. This method works well in comparison to standard methods for filter validation that use hand searching of journals to identify a gold standard, has major advantages in terms of time and resources, and can result in a wider spread of journal titles being included in the gold standard [5]. The following search strategy was applied in MEDLINE and Embase to identify equity-focused systematic reviews.

(equit* or inequal* or inequit* or socioeconomic or socio$economic or dispar*).ti. AND (systematic adj review).ti., limited to English language, excluding conference abstracts

One reviewer searched for reviews published in 2014 and one for reviews published in 2015. Articles were read in full and those that *did not* filter for equity terms were included. Studies of any design that were included in these reviews were considered for inclusion in the gold standard. Equity-focused studies that were excluded in the last screening stage of a systematic review for reasons irrelevant to this current study (e.g. wrong outcome measures, study design not meeting inclusion criteria) were also included.

Second, each reviewer was asked to review their ‘personal files’ (both physical and electronic reference manager databases) to identify all studies of any design reporting an equity outcome.

Third, each reviewer was asked to review at least two issues in any one year (2005 onwards) of the journals *Social Science in Medicine* and *International Journal for Equity in Health* and include all studies reporting an equity outcome.

There were no publication date limits on the gold standard articles. For the first and second strategies the reviewers checked that an identified article was indexed by MEDLINE and/or Embase and excluded articles that were not indexed in either database [5]. Each reviewer was asked to retrieve a minimum of 150 papers.

The MEDLINE citation for each study identified by any of these three methods of retrieval were combined into a single Endnote library and duplicates removed (*n*=5). Duplicates resulted from overlap with the personal files method. Studies were randomly sampled into a validation set (*N*=150), with the remaining randomly split into two evaluation sets (set 1 *N*=101, set 2 *N*=102). This stage was conducted by SG, blind to SLP who compiled the search terms. The sampling process was repeated for the Embase citations; duplicates removed *n*=4; evaluation set 1 *N*=103, set 2 *N*=102; validation set *N*=150.

### Evaluation of the filters

The aim of the evaluation stage was to calculate the sensitivity of the filters against the gold standard evaluation sets [6], examine text and indexing terms for the gold standard studies that were not retrieved, and make any appropriate adjustments to the filters based on these results. The evaluation was first conducted in MEDLINE, and the final strategy subsequently adapted for Embase.

### Validation of the filter

The aim of the validation was to calculate the sensitivity of the evaluated filters against the gold standard validation set. Our validation method is considered an internal gold standard [9]. In order to examine indexing, we also calculated sensitivity for selected subject headings we considered to be general to the social determinants of health.

### Reduction in the number needed to screen

The reduction in the number needed to screen was calculated using search results from the motivating example systematic review in both the MEDLINE and Embase databases. This was defined as the percentage difference in numbers of un-deduplicated records retrieved when conducting the full search with, and without, the equity filter.

### Use of equity filters

We calculated the proportion of all systematic reviews examined in this study that did not use equity filters. We estimated whether the use of filters changed over time with a non-parametric test for trend (‘nptrend’) using Stata V15 (Statacorp LLC).

## RESULTS

### Specific terms

#### Equity search term compilation

We located forty-nine equity-focused systematic reviews published in 2016 and 2017, of which the full text could be retrieved for forty-six (*N*=18 in 2017; *N*=28 in 2016). One of the 2017 reviews and five of the 2016 reviews did not filter for equity, leaving a pool of forty reviews potentially containing details of equity search strategies (*N*=17 in 2017; *N*=23 in 2016).

##### Example search strategies

Thirteen out of the seventeen 2017 reviews (76.4%) that used an equity filter provided an example search strategy for a specific database. A further two listed the concepts searched in the text, and a further two listed examples of keywords.

##### PROGRESS categories populated

Most of the reviews in the initial (2017) search (*N*=12, 70.6%) listed terms that mapped onto the category of socio-economic status; including, for example, the *concepts* of Social welfare, Socio-economic factors, deprivation, and the *measures* of poverty, social class, and income. Five reviews used terms or concepts that did not map onto PROGRESS; insurance, concepts of health disparities, the medically underserved, access and utilisation and we created a separate category for these. Unpopulated or sparsely populated categories were topped up with terms from nine 2016 reviews which contained such terms, and targeted searches located reviews to populate the sparsely populated categories of religion, and social capital; six of which provided terms. Potential equity terms were extracted from strategies reported in a total of 32 different reviews, with between three and 12 different strategies contributing to each PROGRESS category (Table 1).

**Table 1:** Categories populated by terms included in detailed search strategies

PROGRESS heading	No. reviews published in 2017 contributing terms	Further terms needed?	No. reviews published in 2016 contributing terms	Cum. no. reviews contributing terms	Further terms needed?	Reviews included after targeted searches (2012-2015)	Cum. no. reviews contributing terms	Reviews contributing terms
‘Place’ Place of residence	5	No	-	5	No	-	5	[10–14]
‘Race’ Race / ethnicity / culture / language	3	Yes →	6	9	No	-	9	[11, 15–22]
Occupation	6	No	-	6	No	-	6	[11], [13], [16], [23], [24], [25]
‘Gender’ Gender / sex	1	Yes →	6	7	No	-	7	[16], [17], [18], [19], [26], [27], [28]
Religion	0	Yes →	1	1	Yes →	2	3	[17, 29, 30]
Education	7	No	-	7	No	-	7	[11, 13, 16, 24, 25, 31, 32]
‘SES’ Socio-economic status	12	No	-	12	No	-	12	[11], [13], [15], [23], [24], [25], [31], [32], [33], [34], [35], [36]
Social capital	0	Yes →	1	1	Yes →	4	5	[17, 37–40]
Other category not encompassed by PROGRESS-Plus	6	No	-	6	No	-	6	[11, 15, 16, 35, 36, 41]

### Modification of the compiled terms

After inspecting the MeSH browser we noted that the following subject headings were missing from our compiled terms and were added: Social Determinants of Health/; Health Equity/; Psychosocial Deprivation/; Social Stigma/; Working Poor/; Unemployment/; Sociological Factors/; Hierarchy, Social/; exp Educational Status/.

The following keywords and subject headings were considered too general to be of practical use in this filter and were removed: black; asian; Sex/; sex, Women/; women*; woman*; female*; gender; male; men; education; access*; unequal; poor; insurance; gross domestic product; gross national product; gdp; gnp; employment; occupation; profession; and exp Health (nested headings we deemed relevant were added). Terms related to spirituality were deemed irrelevant to disadvantage and removed (spiritual; spirituality; spiritu*). We removed the explosion term from Socioeconomic Factors/ as not all of the nested terms were relevant.

Each search term was entered one per line with terms combined under concepts, and concepts combined overall, by the Boolean ‘OR’ (Additional file 1).

### Non-specific terms

A list of 30 terms and phrases (Additional file 1) relating to the reporting of equity papers were compiled from the abstracts, titles and keywords of the second evaluation set of papers and checked in the first. In the interest of specificity, terms or phrases that retrieved more than 200,000 results with the resulting loss of only 1-2% of evaluation papers were removed. These were: Risk Factors/; associat* with; were high* in; were low* in. Terms that retrieved fewer than 100 results from MEDLINE were also removed. Concepts were combined with the Boolean ‘OR’.

### Gold standard sets

Most of the papers (66.9%) used in the deduplicated gold standard sets were those retrieved from 16 systematic reviews. The personal files method accounted for 20.9% of papers and the hand searching journal method for 12.2%. Publication dates for the articles in the validation sets ranged from 1985 to 2017 with a median publication date of 2009 in the MEDLINE validation set and 2010 in the Embase set.

#### Strategies in OVID

Following testing and modification in the first and then second evaluation sets, the final strategies were run on 02.02.2018 in OVID (MEDLINE, and then Embase). The strategies filtered out between 88-94% of all records (Table 2).

**Table 2:** Percentage of records filtered out

	MEDLINE			Embase		
	No. of records in database^a^	No. retrieved	% filtered out	No. of records in database^a^	No. retrieved	% filtered out
Specific terms	24 442 442	1 598 431	93.5	31 237 516	2 233 531	92.8
Non-specific terms	24 442 442	1 532 739	93.7	31 237 516	1 734 459	94.4
Combined strategy^b^	24 442 442	2 764 686	88.7	31 237 516	3 515 240	88.7

^a^estimated using the search strategy ‘a*.mp.’

^b^The specific terms and non-specific terms strategies combined with Boolean ‘OR’

### Validation results

The combined strategy had the highest sensitivity; over 0.90 in both databases (Table 3). The specific terms strategy had a sensitivity of 0.84 in MEDLINE and 0.87 in Embase. The non-specific terms strategy used alone had relatively low sensitivity.

**Table 3:** Sensitivity of finalised strategy against validation set

	MEDLINE			Embase		
	No. in set	No. retrieved	Sensitivity (95% CI)	No. in set	No. retrieved	Sensitivity (95% CI)
Specific terms	150	126	0.84 (0.77, 0.89)	150	131	0.87 (0.81, 0.92)
Non-specific terms	150	99	0.66 (0.57, 0.74)	150	97	0.65 (0.56, 0.72)
Combined strategy^a^	150	138	0.92 (0.86, 0.96)	150	141	0.94 (0.89, 0.97)

*CI* binomial confidence interval

^a^The specific terms and non-specific terms strategies combined with Boolean ‘OR’

For the specific terms strategy, we sought to evaluate the extent to which studies were indexed on subject headings. As the denominator we counted only those papers published in the year following the introduction of the heading, or subsequent years. The subject heading most obviously related to the social determinants of health were established in MEDLINE and Embase in only 2014. We were unable to evaluate these headings because only six studies in our evaluation sets were published in or after 2015. None were indexed on the heading (Table 4). The second heading was related to health inequalities, established in Embase in 2009 and MEDLINE in 2008. Sensitivity was low; 0.18 in MEDLINE and 0.13 in Embase. We could not evaluate the effect of the subject heading ‘Health equity’ as it was only introduced in 2016 in both databases.

**Table 4:** Sensitivity of selected subject headings against validation set

Database	Subject heading	No. in set^a^	No. retrieved	Sensitivity (95% CI)
MEDLINE	Social determinants of Health/	1	0	-
	Health Status Disparities/	87	16	0.18 (0.10, 0.28)
Embase	"social determinants of health"/	5	0	-
	health disparity/	77	10	0.13 (0.06, 0.23)

*CI* binomial confidence interval

^a^published, at the earliest, in the year following the subject heading introduction

Full strategies including line numbers are found in the appendix.

### Reduction in the number needed to screen

We added the filters (Boolean ‘AND’) to the search strategy developed for motivating example systematic review. This aimed to locate interventions reporting effects on mental health inequalities. The percentage reduction in the number needed to screen (NNS) are presented in Table 5. The specific terms strategy reduced the NNS by around 77% in both databases. The combined strategy reduced the NNS by 59.7% in MEDLINE, and 63.5% in Embase.

**Table 5:** Reduction in the number needed to screen

	MEDLINE			
	NNS^a^, N	Reduction in NNS, n (%)	NNS^a^, N	Reduction in NNS, n (%)
(1) Unfiltered strategy	14 452	-	26 552	-
(2) (1) plus specific terms	3 203	11 249 (77.8)	6 092	20 460 (77.1)
(3) (1) plus non-specific terms	4 193	10 259 (70.9)	6 081	20 471 (77.1)
(4) (1) plus combined strategy	5 824	8 628 (59.7)	9 690	16 862 (63.5)

*NNS* number need to screen

^a^Not de-duplicated

### Use of equity filters

One hundred and eighteen equity-focused systematic reviews, located in MEDLINE and Embase, were examined in this study. Seventy-two (published in years 2014 & 2015) were located as a strategy to find gold standard records, and 46 (2016 & 2017) were located in order to compile search terms. Out of the 118 reviews, only 22 (18.6%) did not use an equity filter (Table 6). There was little evidence of change in the use of filters over the four publication years; *z* = -1.48, *p*=0.139.

**Table 6:** Use of equity filters in systematic reviews

	Year of publication				
	2014	2015	2016	2017	All years
Systematic reviews identified, N	27	45	28	18	118
Systematic reviews that did not use an equity filter, n (%)	6 (22.2)	10 (22.2)	5 (17.9)	1 (5.5)	22 (18.6)

## Discussion

We aimed to develop and test a sensitive search filter to locate studies reporting equity outcomes in MEDLINE and Embase. A comprehensive strategy comprised of specific terms related to the social determinants of health filtered out 93-95% of all records, and had a sensitivity of 0.84 in MEDLINE, and 0.87 in Embase against a gold standard set of records. The sensitivity improved to 0.92 in MEDLINE (0.94 in Embase) when combined with a strategy comprising phrases often used to report equity study aims or findings. This combination met our criteria for a ‘good’ filter. The number needed to screen for the motivating systematic review was reduced by 77% by applying the specific terms strategy, and by 59.7% (MEDLINE) to 63.5% (Embase) by applying the combined strategy. Despite our study being the first to report the development and testing of an equity filter, we found that eighty-one per cent of systematic reviews published between 2014 and 2017 applied apparently unevaluated filters as part of their search strategy.

We used a combination of methods to collect gold standard papers; references from systematic reviews, personal files and hand searching journals. While combining these methods is recommended [9], , it may not eliminate bias caused by any one method. Accordingly, ur gold standard sample may not be fully representative of those in the corpus of literature, particularly studies where the inequality is not indexed and reported only in the body of the text. The majority of citations were identified from papers included in equity-focused systematic reviews that did not apply a filter; i.e. some of these studies may have been identified as reporting an equity outcome only after reading the whole article. Given that the most challenging papers to identify are those that provide few clues in the title, abstract, or keywords that they report equity outcomes, the over-inclusion of such studies improves the rigour of our gold standard set. However, studies contained in the reviews we examined may not be representative of the full corpus of literature and important studies may be missed [5]. Further, the reviews we located may not themselves fully represent the sample universe of equity-focused reviews [9]. We hand-searched only a limited number of issues in two journals, and records contained in personal files are unlikely to have broad coverage of the topic area. We used the same method to develop and measure the performance of our sample (internal validation). Validation of an external set, located by different methods than those used for the evaluation, would have strengthened our study [9] but this was beyond current resources. Although we did not formally assess reliability, two authors experienced in social epidemiology independently identified gold standard studies. There were few duplicates in the studies they identified. The selection and randomisation of gold standard studies into evaluation and validation sets was carried out independent of the author who compiled specific terms strategy, and prior to this stage being carried out. Gold standard study selection was also carried out blind to the search term compilation, minimising the risk of purposive sampling.

We used PROGRESS to help structure the specific terms strategy. Terms were mapped onto what we considered to be the most relevant concepts, but this process is subjective. Some terms cover more than one concept; for example ‘Social determinants of health’ apply to all categories and there is overlap between terms relating to SES and social capital, and differences in these between MEDLINE and Embase. We caution authors interested in filtering for a single concept to scrutinise other concepts for related terms, and emphasise that we did not estimate performance for single concepts. No -Plus terms were used for the specific terms strategy, which means that this filter may perform poorly if determinants such as age-related inequalities, disabilities or other personal characteristics are of interest. Similarly, terms such as ‘insurance’ are missing, which may disproportionately bias against US research.

Further reductions in the number needed to screen could be achieved by using text mining. Text mining techniques have been found to be useful for helping devise search strategies for complex topics and for helping to rank order records to help with screening large libraries of records. Text mining encompasses a range of statistical approaches to textual analysis and much of its value can lie in its automation and objectivity. Procedures to develop search strategies routinely using text mining approaches are available [42–44] and these tend to focus on frequency analysis of words and phrases within records. Another approach is to generate large libraries of records and then use text mining for study identification [45]. Text mining technologies can prioritise title-abstract records for manual screening, use active learning to improve the prioritisation as more records are screened and stopping rules. Given the complexity of this topic and the potential for large numbers of records, text mining could prove particularly useful in this area.

To our knowledge, our filters are the first to be formally assessed for performance; but consequent to this first attempt, they are basic and lengthy. Further development could include refining to improve sensitivity and specificity, screening all retrieved records for equity studies in order to calculate accuracy and precision, and validating against an external standard [9].

We surmise that there is a need for validated equity filters. We found the vast majority of equity-focused systematic reviews applied a filter, presumably to reduce the burden of screening for inclusion. There were, however, no reports that any equity strategy had been formally tested. Most of the search strategies in these reviews would have been compiled prior to guidance (not to filter) issued in 2015 [4], but it is unclear whether researchers will follow this guidance given the large, and ever increasing, numbers needed to screen. This is perhaps particularly true for reviews without substantial topic focus such as the effect of social determinants on a particular condition, or range of conditions, where a vast literature might be retrieved.

We chose to evaluate a broad social determinants of health filter, and consequently our study was not powered to determine the performance of the filter on, for example, studies that reported outcomes by gender, or employment status. We evaluated two subject heading terms that could be used by indexers to categorise equity studies more broadly, and not just on the determinant measured, but found very low levels of indexing. We suspect that this is due to a combination of two factors; the relatively recent introduction of these terms and a lack of familiarity of indexers with the concept of the social determinants of health, and, consequently, what equity studies look like. In particular, this under-indexing may be amplified in the many studies where equity is not the focus, but reported as a secondary outcome. Authors of studies that report equity outcomes (whether primarily or secondarily) could assist in this process by consistently suggesting the keywords ‘Social determinants of health’ and/or ‘Health inequality’ in their manuscript, along with the specific determinants measured. The Consolidated Standards of Reporting Trials (CONSORT) equity extension for randomised trials recommends trialists suggest their papers are indexed under the heading ‘Health equity’ [46]. The usefulness of indexing on any of these will only be realised for the inclusion of current and ongoing primary research in future reviews as all three of them have only recently been added as subject headings (2016 in the case of Health equity) and existing studies are not retrospectively re-indexed. The term ‘health equity’ is recommended to be included in titles of trials where appropriate [46]; we suggest that the word ‘equity’ is sufficient for this purpose and any of these phrases could be usefully incorporated into abstracts which would then be picked up with key word searches using the multi-purpose (.mp.) field.

## Conclusions

The highest sensitivity in retrieving studies reporting outcomes by equity was a combined approach of a comprehensive list of specific terms and subject headings related to social and economic factors that produce health inequality, plus a list of non-specific terms related to the reporting of equity studies. At this early stage of equity filter development, if systematic reviewers wish to filter, this combined approach is recommended. Further efforts should concentrate on the standardisation of indexing for equity studies and additional development and testing of both specific and non-specific terms for accurate study retrieval.

## Additional file

Additional file 1: Search strategies and validation set papers. Contains validated search strategies, the number of records retrieved, and the MEDLINE validation set of papers. (PDF 722 kb)

## Abbreviations

CONSORT: Consolidated Standards of Reporting Trials
MeSH: Medical Subject Headings
NNS: Number needed to screen

## References

[1] Attwood S, van Sluijs E, Sutton S (2016). Exploring equity in primary-care-based physical activity interventions using PROGRESS-Plus: a systematic review and evidence synthesis. Int J Behav Nutr Phys Act.

[2] Whitehead M (2007). A typology of actions to tackle social inequalities in health. J Epidemiol Community Health.

[3] Welch VA, Petticrew M, O’Neill J, Waters E, Armstrong R, Bhutta ZA, Francis D, Koehlmoos TP, Kristjansson E, Pantoja T, Tugwell P (2013). Health equity: evidence synthesis and knowledge translation methods. System Rev.

[4] Welch V, Petticrew M, Petkovic J, Moher D, Waters E, White H, Tugwell P (2015). Extending the PRISMA statement to equity-focused systematic reviews (PRISMA-E 2012): explanation and elaboration. Int J Equity Health.

[5] Sampson M, Zhang L, Morrison A, Barrowman NJ, Clifford TJ, Platt RW, Klassen TP, Moher D (2006). An alternative to the hand searching gold standard: validating methodological search filters using relative recall. BMC Med Res Methodol.

[6] Jenkins M (2004). Evaluation of methodological search filters—a review. Health Inf Libr J.

[7] Vincent S, Greenley S, Beaven O (2003). Clinical Evidence diagnosis: Developing a sensitive search strategy to retrieve diagnostic studies on deep vein thrombosis: a pragmatic approach. Health Inf Libr J.

[8] Olivers S, Kavanagh J, Caird J, Lorenc T, Oliver K, Harden A, Thomas J, Greaves A, Oakley A (2008). Health promotion, inequalities and young people’s health: a systematic review of research.

[9] Lefebvre C, Glanville J, Beale S, Boachie C, Duffy S, Fraser C, Harbour J, McCool R, Smith L (2017). Assessing the performance of methodological search filters to improve the efficiency of evidence information retrieval: five literature reviews and a qualitative study. Health Technol Assess.

[10] Alston L, Allender S, Peterson K, Jacobs J, Nichols M (2017). Rural Inequalities in the Australian Burden of Ischaemic Heart Disease: A Systematic Review. Heart Lung Circ.

[11] Andrea SB, Hooker ER, Messer LC, Tandy T, Boone-Heinonen J. Does the association between early life growth and later obesity differ by race/ethnicity or socioeconomic status? A systematic review. Ann Epidemiol. 2017. 10.1016/j.annepidem.2017.08.019 PMC6688753 28911983

[12] Danielewicz AL, dos Anjos JC, Bastos JL, Boing AC, Boing AF (2017). Association between socioeconomic and physical/built neighborhoods and disability: A systematic review. Prev Med.

[13] Maddock J, Wulaningsih W, Hardy R. Impact of body size, nutrition and socioeconomic position in early life on the epigenome: A systematic review protocol. System Rev. 2017;6(1) (no pagination). 10.1186/s13643-017-0521-8 PMC5499029 28679412

[14] Vasquez-Vera H, Palencia L, Magna I, Mena C, Neira J, Borrell C (2017). The threat of home eviction and its effects on health through the equity lens: A systematic review. Soc Sci Med.

[15] Pratt CA, Loria CM, Arteaga SS, Nicastro HL, Lopez-Class M, de Jesus JM, Srinivas P, Maric-Bilkan C, Schwartz Longacre L, Boyington JEA (2017). A Systematic Review of Obesity Disparities Research. Am J Prev Med.

[16] Sun EY, Jadotte YT, Halperin W (2017). Disparities in cardiac rehabilitation participation in the United States: A systematic review and meta-analysis. J Cardiopulm Rehab Prev.

[17] Campos-Matos I, Russo G, Perelman J. Connecting the dots on health inequalities - A systematic review on the social determinants of health in Portugal. Int J Equity Health. 2016;15(1) (no pagination). 10.1186/s12939-016-0314-z PMC4754837 26879973

[18] Carter A, Corbelli J, Borrero S, Wessel C, Washington DL, Bean-Mayberry B, Batch BC, Breland JY, DiLeone B, Foynes MM (2016). Racial and Ethnic Health Care Disparities Among Women in the Veterans Affairs Healthcare System: A Systematic Review. Womens Health Issues.

[19] Santos Salas A, Fuentes Contreras J, Armijo-Olivo S, Saltaji H, Watanabe S, Chambers T, Walter L, Cummings GG (2016). Non-pharmacological cancer pain interventions in populations with social disparities: a systematic review and meta-analysis. Support Care Cancer.

[20] Segal N, Greenberg D, Dagan R, Ben-Shimol S (2016). Disparities in PCV impact between different ethnic populations cohabiting in the same region: A systematic review of the literature. Vaccine.

[21] Smith SK, Sousa MS, Essink-Bot ML, Halliday J, Peate M, Fransen M (2016). Socioeconomic Differences in Informed Decisions About Down Syndrome Screening: A Systematic Review and Research Agenda. J Health Commun.

[22] Youl PH, Dasgupta P, Youlden D, Aitken JF, Garvey G, Zorbas H, Chynoweth J, Wallington I, Baade PD (2016). A systematic review of inequalities in psychosocial outcomes for women with breast cancer according to residential location and Indigenous status in Australia. Psycho-Oncology.

[23] Allen L, Williams J, Townsend N, Mikkelsen B, Roberts N, Foster C, Wickramasinghe K (2017). Socioeconomic status and non-communicable disease behavioural risk factors in low-income and lower-middle-income countries: a systematic review. Lancet Glob Health.

[24] de Mestral C, Mayen AL, Petrovic D, Marques-Vidal P, Bochud M, Stringhini S (2017). Socioeconomic Determinants of Sodium Intake in Adult Populations of High-Income Countries: A Systematic Review and Meta-Analysis. Am J Public Health.

[25] Hyun KK, Brieger D, Woodward M, Richtering S, Redfern J. The effect of socioeconomic disadvantage on prescription of guideline-recommended medications for patients with acute coronary syndrome: Systematic review and meta-analysis. Int J Equity Health. 2017;16(1) (no pagination). 10.1186/s12939-017-0658-z PMC5579970 28859658

[26] Boodhoo Kamini Devi, Liu Sijia, Zuo Xiaoxia (2016). Impact of sex disparities on the clinical manifestations in patients with systemic lupus erythematosus. Medicine.

[27] Huang FY, Huang BT, Wang PJ, Zhang C, Zuo ZL, Liao YB, Xia TL, Gui YY, Peng Y, Liu W (2016). Gender Disparity in the Safety and Efficacy of Radial and Femoral Access for Coronary Intervention: A Systematic Review and Meta-Analysis. Angiology.

[28] McCollum R, Gomez W, Theobald S, Taegtmeyer M (2016). How equitable are community health worker programmes and which programme features influence equity of community health worker services? A systematic review. BMC Public Health.

[29] Agli O, Bailly N, Ferrand C (2015). Spirituality and religion in older adults with dementia: A systematic review. Int Psychogeriatr.

[30] Schreiber JA, Brockopp DY (2012). Twenty-five years later-what do we know about religion/spirituality and psychological well-being among breast cancer survivors? A systematic review. J Cancer Surviv.

[31] Almeida APSC, Nunes BP, Duro SMS, Facchini LA (2017). Socioeconomic determinants of access to health services among older adults: a systematic review. Rev Saude Publica.

[32] Gebremariam MK, Lien N, Nianogo RA, Arah OA (2017). Mediators of socioeconomic differences in adiposity among youth: a systematic review. Obes Rev.

[33] Forrest LF, Sowden S, Rubin G, White M, Adams J (2017). Socio-economic inequalities in stage at diagnosis, and in time intervals on the lung cancer pathway from first symptom to treatment: Systematic review and meta-analysis. Thorax.

[34] Ribeiro WS, Bauer A, Andrade MCR, York-Smith M, Pan PM, Pingani L, Knapp M, Coutinho ESF, Evans-Lacko S (2017). Income inequality and mental illness-related morbidity and resilience: a systematic review and meta-analysis. Lancet Psychiatry.

[35] Salway SM, Payne N, Rimmer M, Buckner S, Jordan H, Adams J, Walters K, Sowden SL, Forrest L, Sharp L, et al. Identifying inequitable healthcare in older people: Systematic review of current research practice. Int J Equity Health. 2017;16(1) (no pagination). 10.1186/s12939-017-0605-z PMC5505033 28697768

[36] Scott Anne, Chambers Duncan, Goyder Elizabeth, O’Cathain Alicia (2017). Socioeconomic inequalities in mortality, morbidity and diabetes management for adults with type 1 diabetes: A systematic review. PLOS ONE.

[37] Agampodi TC, Agampodi SB, Glozier N, Siribaddana S (2015). Measurement of social capital in relation to health in low and middle income countries (LMIC): A systematic review. Soc Sci Med.

[38] Choi M, Mesa-frias M, Nuesch E, Hargreaves J, Prieto-merino D, Bowling A, Smith GD, Ebrahim S, Dale CE, Casas JP (2014). Social capital, mortality, cardiovascular events and cancer: A systematic review of prospective studies. Int J Epidemiol.

[39] Ehsan AM, De Silva MJ (2015). Social capital and common mental disorder: a systematic review. J Epidemiol Community Health.

[40] Uphoff Eleonora P, Pickett Kate E, Cabieses Baltica, Small Neil, Wright John (2013). A systematic review of the relationships between social capital and socioeconomic inequalities in health: a contribution to understanding the psychosocial pathway of health inequalities. International Journal for Equity in Health.

[41] Simoni JM, Smith L, Oost KM, Lehavot K, Fredriksen-Goldsen K (2017). Disparities in Physical Health Conditions Among Lesbian and Bisexual Women: A Systematic Review of Population-Based Studies. J Homosex.

[42] Hausner E, Waffenschmidt S, Kaiser T, Simon M (2012). Routine development of objectively derived search strategies. Syst Rev.

[43] Hausner E, Guddat C, Hermanns T, Lampert U, Waffenschmidt S (2015). Development of search strategies for systematic reviews: validation showed the noninferiority of the objective approach. J Clin Epidemiol.

[44] EUnetHTA (2017). Guideline: Process of information retrieval for systematic reviews and health technology assessments on clinical effectiveness.

[45] Shemilt I, Simon A, Hollands GJ, Marteau TM, Ogilvie D, O'Mara-Eves A, Kelly MP, Thomas J (2014). Pinpointing needles in giant haystacks: use of text mining to reduce impractical screening workload in extremely large scoping reviews. Res Synth Methods.

[46] Welch VA, Norheim OF, Jull J, Cookson R, Sommerfelt H, Tugwell P. CONSORT-Equity 2017 extension and elaboration for better reporting of health equity in randomised trials. BMJ. 2017;**359**. 10.1136/bmj.j5085 29170161

