# Supplementary material for: Development and validation of a search filter to identify equity-focused studies: reducing the number needed to screen
Source: BMC Med Res Methodol. 2018 Oct 12;18:106. doi: 10.1186/s12874-018-0567-x (PMC6186133; doi:10.1186/s12874-018-0567-x)
Supplement: Supplementary file 1 — Search strategies and validation set papers. Contains validated search strategies, the number of records retrieved, and the MEDLINE validation set of papers. (PDF 722 kb) [file 12874_2018_567_MOESM1_ESM.pdf]

# Development and validation of a search filter to identify equity-focused studies: reducing the number needed to screen

Stephanie L. Prady, Eleonora P. Uphoff, Madeleine Power, Su Golder

## Search strategies and validation set papers

## Final strategies and number of retrieved records

All final strategies were run on 2.2.2018.

| **MEDLINE specific terms** | | |
|---|---|---|
| 1 | Residence Characteristics/ | 28,899 |
| 2 | Environment design/ | 5,307 |
| 3 | exp Marital status/ | 35,112 |
| 4 | neighbo?rhood*.mp. | 20,493 |
| 5 | residential environment*.mp. | 485 |
| 6 | rural*.mp. | 128,713 |
| 7 | inner?city.mp. | 49 |
| 8 | housing instability.mp. | 129 |
| 9 | housing insecurity.mp. | 35 |
| 10 | housing strain.mp. | 4 |
| 11 | housing security.mp. | 17 |
| 12 | mortgage problems.mp. | 0 |
| 13 | foreclosure.mp. | 129 |
| 14 | eviction*.mp. | 538 |
| 15 | housing loss.mp. | 10 |
| 16 | home repossession*.mp. | 2 |
| 17 | home ownership.mp. | 327 |
| 18 | (repossess* adj3 hous*).mp. | 5 |
| 19 | (repossess* adj3 propert*).mp. | 1 |
| 20 | mortgage delinquency.mp. | 6 |
| 21 | mortgage arrears.mp. | 1 |
| 22 | mortgage debt*.mp. | 3 |
| 23 | overcrowding.mp. | 2,069 |
| 24 | (living adj1 (outside or inside or near* or adjacent)).mp. | 2,546 |
| 25 | (household adj2 size).mp. | 899 |
| 26 | (marital status or marriage status).mp. | 21,064 |
| 27 | (widow* or cohabit* or divorce* or single parent* or live* alone).mp. | 19,588 |
| 28 | or/1-27 | 229,913 |
| 29 | Cultural Deprivation/ | 1,122 |
| 30 | Acculturation/ | 5,764 |
| 31 | Culture/ | 30,684 |
| 32 | Cross-Cultural Comparison/ | 23,908 |
| 33 | Cultural Characteristics/ | 15,147 |
| 34 | Cultural Diversity/ | 10,449 |
| 35 | Language/ | 35,612 |
| 36 | "Transients and Migrants"/ | 9,888 |
| 37 | exp "Emigrants and Immigrants"/ | 9,505 |
| 38 | Minority groups/ | 12,197 |
| 39 | Minority health/ | 609 |

| MEDLINE specific terms | | |
|---|---|---|
| 40 | Prejudice/ | 23,766 |
| 41 | Racism/ | 1,336 |
| 42 | Xenophobia/ | 20 |
| 43 | Social Discrimination/ | 725 |
| 44 | exp Race Relations/ | 3,672 |
| 45 | exp Ethnic Groups/ | 136,459 |
| 46 | exp Continental Population Groups/ | 194,168 |
| 47 | Refugees/ | 8,335 |
| 48 | minorit*.mp. | 55,472 |
| 49 | migration background.mp. | 273 |
| 50 | racial.mp. | 29,233 |
| 51 | racism.mp. | 3,105 |
| 52 | ethnology.mp. | 1,701 |
| 53 | race.mp. | 78,915 |
| 54 | ethnic*.mp. | 138,693 |
| 55 | non?English.mp. | 16 |
| 56 | language other than.mp. | 268 |
| 57 | latino*.mp. | 8,646 |
| 58 | latina*.mp. | 3,026 |
| 59 | hispanic*.mp. | 43,522 |
| 60 | whites.mp. | 21,682 |
| 61 | caucasian*.mp. | 47,538 |
| 62 | non?white.mp. | 2,251 |
| 63 | Torres Strait Islander.mp. | 861 |
| 64 | aboriginal.mp. | 6,605 |
| 65 | native american.mp. | 2,868 |
| 66 | inuit.mp. | 1,431 |
| 67 | eskimo.mp. | 655 |
| 68 | first nation*.mp. | 3,225 |
| 69 | indigenous.mp. | 23,452 |
| 70 | english as a second language.mp. | 305 |
| 71 | foreign language.mp. | 635 |
| 72 | or/29-71 | 573,055 |
| 73 | Occupations/ | 21,771 |
| 74 | Unemployment/ | 6,164 |
| 75 | occupations.mp. | 39,535 |
| 76 | unemployment.mp. | 11,136 |
| 77 | or/73-76 | 50,296 |
| 78 | exp Gender Identity/ | 17,685 |
| 79 | Women's Health/ | 25,304 |
| 80 | gender differences.mp. | 20,416 |
| 81 | (sex disparit* or sex difference?).mp. | 25,907 |
| 82 | gender identity.mp. | 17,497 |
| 83 | sex role.mp. | 1,423 |
| 84 | wom#n* role?.mp. | 494 |
| 85 | m#n* role?.mp. | 8,188 |
| 86 | gender* role?.mp. | 2,095 |
| 87 | servicewomen.mp. | 68 |
| 88 | Sex factors/ | 240,269 |
| 89 | or/78-88 | 313,465 |
| 90 | exp Educational status/ | 46,834 |
| 91 | Education/ | 19,636 |
| 92 | Schooling.mp. | 5,759 |

| MEDLINE specific terms | | |
|---|---|---|
| 93 | educational status.mp. | 48,579 |
| 94 | (education* adj2 level?).mp. | 33,184 |
| 95 | ((higher or better or worse or less) adj educated).mp. | 4,276 |
| 96 | ((higher or better or worse or less) adj level? of education).mp. | 1,495 |
| 97 | or/90-96 | 97,846 |
| 98 | Religion/ | 13,229 |
| 99 | religi*.mp. | 51,772 |
| 100 | or/98-99 | 51,772 |
| 101 | Social determinants of Health/ | 1,328 |
| 102 | Psychosocial Deprivation/ | 1,920 |
| 103 | Sociological Factors/ | 325 |
| 104 | Working Poor/ | 10 |
| 105 | Hierarchy, Social/ | 2,015 |
| 106 | disparit*.mp. | 57,213 |
| 107 | inequalit*.mp. | 21,501 |
| 108 | inequit*.mp. | 5,889 |
| 109 | equity.mp. | 9,641 |
| 110 | deprivation.mp. | 70,377 |
| 111 | gini.mp. | 742 |
| 112 | concentration index.mp. | 793 |
| 113 | Socioeconomic Factors/ | 137,422 |
| 114 | Social Welfare/ | 8,806 |
| 115 | exp Social Class/ | 37,022 |
| 116 | exp Poverty/ | 37,272 |
| 117 | Income/ | 25,581 |
| 118 | Social class*.mp. | 41,202 |
| 119 | social determinants.mp. | 4,250 |
| 120 | social status.mp. | 4,516 |
| 121 | social position.mp. | 786 |
| 122 | social background.mp. | 993 |
| 123 | social circumstance*.mp. | 980 |
| 124 | socio-economic.mp. | 22,017 |
| 125 | socioeconomic.mp. | 176,022 |
| 126 | sociodemographic.mp. | 28,750 |
| 127 | socio-demographic.mp. | 15,511 |
| 128 | SES.mp. | 14,857 |
| 129 | disadvantaged.mp. | 9,148 |
| 130 | impoverished.mp. | 2,662 |
| 131 | poverty.mp. | 47,271 |
| 132 | economic level.mp. | 759 |
| 133 | assets index.mp. | 17 |
| 134 | income*.mp. | 95,525 |
| 135 | or/101-134 | 467,331 |
| 136 | Social Stigma/ | 4,814 |
| 137 | social capital/ | 451 |
| 138 | Social Control, Informal/ | 3,590 |
| 139 | exp Social Support/ | 62,350 |
| 140 | exp Social Environment/ | 104,543 |
| 141 | Trust/ | 7,820 |
| 142 | Social conditions/ | 9,036 |
| 143 | Social isolation/ | 12,167 |
| 144 | Social marginalization/ | 268 |
| 145 | Anomie/ | 428 |

| **MEDLINE specific terms** | | |
|---|---|---|
| 146 | social participation/ | 1,516 |
| 147 | social exclusion.mp. | 1,080 |
| 148 | (social adj (capital or cohes* or organis* or organiz*)).mp. | 4,667 |
| 149 | (community adj3 (cohes* or participa*)).mp. | 27,025 |
| 150 | ((neighbourhood or neighborhood) adj cohes*).mp. | 88 |
| 151 | social relationships.mp. | 3,676 |
| 152 | social network*.mp. | 11,532 |
| 153 | collective efficacy.mp. | 283 |
| 154 | civil society.mp. | 1,095 |
| 155 | informal social control.mp. | 69 |
| 156 | neighbo*rhood disorder.mp. | 121 |
| 157 | social disorgani?ation.mp. | 147 |
| 158 | anomie.mp. | 510 |
| 159 | social support.mp. | 72,250 |
| 160 | social participation.mp. | 2,846 |
| 161 | trust.mp. | 25,594 |
| 162 | emotional support.mp. | 4,191 |
| 163 | psychosocial support.mp. | 2,279 |
| 164 | community capital.mp. | 4 |
| 165 | neighbo*rhood cohesion.mp. | 85 |
| 166 | social influence.mp. | 1,303 |
| 167 | (soci*context* or soci*-context*).mp. | 7,465 |
| 168 | or/136-167 | 212,146 |
| 169 | Health Status Disparities/ | 11,609 |
| 170 | Health Services Accessibility/ | 63,492 |
| 171 | Health Equity/ | 352 |
| 172 | health*care disparit*.mp. | 12,500 |
| 173 | health care disparit*.mp. | 761 |
| 174 | health status disparit*.mp. | 11,683 |
| 175 | health disparit*.mp. | 6,836 |
| 176 | health inequalit*.mp. | 3,488 |
| 177 | health inequit*.mp. | 972 |
| 178 | medically underserved.mp. | 7,030 |
| 179 | or/169-178 | 92,642 |
| 180 | 28 or 72 or 77 or 89 or 97 or 100 or 135 or 168 or 179 | 1,598,431 |

| **MEDLINE non-specific terms** | | |
|---|---|---|
| 1 | potential determinants.mp. | 1,274 |
| 2 | significant correlates of.mp. | 903 |
| 3 | (independent correlates or independent association*).mp. | 7,649 |
| 4 | variables associated with.mp. | 7,968 |
| 5 | determinants of.mp. | 119,923 |
| 6 | factors associated with.mp. | 80,220 |
| 7 | identif* determinants.mp. | 1,431 |
| 8 | (more likely or less likely or just as likely).mp. | 203,709 |
| 9 | risk factors for.mp. | 829,444 |
| 10 | (significantly related to or significant predictor).mp. | 41,608 |
| 11 | (also adj2 associated with).mp. | 45,598 |
| 12 | (at increased risk or at decreased risk).mp. | 26,633 |
| 13 | association* between.mp. | 343,116 |
| 14 | (positively associated or negatively associated).mp. | 39,113 |
| 15 | differed by.mp. | 8,713 |

| **MEDLINE non-specific terms** | | |
|---|---|---|
| 16 | (were high* amongst or were low* amongst).mp. | 776 |
| 17 | (inverse relationship with or inversely associated with or inversely related to).mp. | 31,681 |
| 18 | reverse association.mp. | 135 |
| 19 | differentially affects.mp. | 1,790 |
| 20 | evidence of a link between.mp. | 1,207 |
| 21 | (significantly adj3 likelihood of).mp. | 1,803 |
| 22 | protective factors for.mp. | 7,974 |
| 23 | (differ* adj2 according to).mp. | 19,676 |
| 24 | (inverse adj2 gradient).mp. | 181 |
| 25 | (positive adj2 gradient).mp. | 331 |
| 26 | (negative adj2 gradient).mp. | 252 |
| 27 | (trends were adj3 across).mp. | 833 |
| 28 | (related to adj3 variable*).mp. | 15,782 |
| 29 | (differences were adj3 explained by).mp. | 6,285 |
| 30 | (significant among or no# significant among).mp. | 2,233 |
| 31 | or/1-30 | 1,532,739 |

| **MEDLINE combined strategy** | |
|---|---|
| [Specific strategy line] 180 OR [non-specific strategy line] 31 | 2 764,686 |

| **Embase specific terms** | | |
|---|---|---|
| 1 | demography/ | 181,764 |
| 2 | environmental planning/ | 9,448 |
| 3 | marriage/ | 50,904 |
| 4 | divorce/ | 6,999 |
| 5 | cohabitation/ | 574 |
| 6 | widow/ | 2,099 |
| 7 | exp "single (marital status)"/ | 5,124 |
| 8 | neighbo?rhood*.mp. | 28,378 |
| 9 | residential environment*.mp. | 680 |
| 10 | rural*.mp. | 160,984 |
| 11 | inner?city.mp. | 143 |
| 12 | housing instability.mp. | 210 |
| 13 | housing insecurity.mp. | 56 |
| 14 | housing strain.mp. | 4 |
| 15 | housing security.mp. | 24 |
| 16 | mortgage problems.mp. | 1 |
| 17 | foreclosure.mp. | 190 |
| 18 | eviction*.mp. | 867 |
| 19 | housing loss.mp. | 17 |
| 20 | home repossession*.mp. | 2 |
| 21 | home ownership.mp. | 403 |
| 22 | (repossess* adj3 hous*).mp. | 5 |
| 23 | (repossess* adj3 propert*).mp. | 1 |
| 24 | mortgage delinquency.mp. | 6 |
| 25 | mortgage arrears.mp. | 1 |
| 26 | mortgage debt*.mp. | 5 |
| 27 | overcrowding.mp. | 3,055 |
| 28 | (living adj1 (outside or inside or near* or adjacent)).mp. | 3,514 |

| Embase specific terms | | |
|---|---|---|
| 29 | (household adj2 size).mp. | 1,175 |
| 30 | (marital status or marriage status).mp. | 24,519 |
| 31 | (widow* or cohabit* or divorce* or single parent* or live* alone).mp. | 27,354 |
| 32 | or/1-31 | 441,524 |
| 33 | exp cultural deprivation/ | 1,073 |
| 34 | cultural factor/ | 56,462 |
| 35 | cultural anthropology/ | 51,865 |
| 36 | cultural diversity/ | 668 |
| 37 | exp migrant/ | 28,585 |
| 38 | minority group/ | 13,525 |
| 39 | minority health/ | 580 |
| 40 | prejudice/ | 1,897 |
| 41 | exp social discrimination/ | 13,193 |
| 42 | exp race relation/ | 4,383 |
| 43 | exp ethnic group/ | 118,329 |
| 44 | exp ancestry group/ | 244,999 |
| 45 | exp refugee/ | 10,302 |
| 46 | minorit*.mp. | 80,900 |
| 47 | migration background.mp. | 475 |
| 48 | racial.mp. | 67,137 |
| 49 | racism.mp. | 5,689 |
| 50 | ethnology.mp. | 72,429 |
| 51 | race.mp. | 166,582 |
| 52 | ethnic*.mp. | 241,363 |
| 53 | non?English.mp. | 27 |
| 54 | language other than.mp. | 408 |
| 55 | latino*.mp. | 13,368 |
| 56 | latina*.mp. | 4,270 |
| 57 | hispanic*.mp. | 71,944 |
| 58 | whites.mp. | 29,995 |
| 59 | caucasian*.mp. | 137,633 |
| 60 | non?white.mp. | 2,861 |
| 61 | Torres Strait Islander.mp. | 1,398 |
| 62 | aboriginal.mp. | 9,302 |
| 63 | native american.mp. | 4,250 |
| 64 | inuit.mp. | 2,073 |
| 65 | eskimo.mp. | 2,869 |
| 66 | first nation*.mp. | 4,950 |
| 67 | indigenous.mp. | 33,196 |
| 68 | english as a second language.mp. | 1,086 |
| 69 | foreign language.mp. | 908 |
| 70 | or/33-69 | 801,788 |
| 71 | exp employment status/ | 26,211 |
| 72 | job characteristics/ | 552 |
| 73 | occupations.mp. | 13,814 |
| 74 | unemployment.mp. | 19,534 |
| 75 | or/71-74 | 43,593 |
| 76 | exp gender identity/ | 14,693 |
| 77 | women's health/ | 23,875 |
| 78 | sex difference/ | 347,261 |
| 79 | (sex disparit* or sex difference?).mp. | 356,593 |
| 80 | gender identity.mp. | 15,819 |
| 81 | sex role.mp. | 5,505 |

| Embase specific terms | | |
|---|---|---|
| 82 | wom#n* role?.mp. | 532 |
| 83 | m#n* role?.mp. | 10,505 |
| 84 | gender* role?.mp. | 2,873 |
| 85 | servicewomen.mp. | 82 |
| 86 | or/76-85 | 407,453 |
| 87 | exp educational status/ | 60,655 |
| 88 | schooling.mp. | 7,964 |
| 89 | educational status.mp. | 61,781 |
| 90 | (education* adj2 level?).mp. | 52,239 |
| 91 | ((higher or better or worse or less) adj educated).mp. | 5,543 |
| 92 | ((higher or better or worse or less) adj level? of education).mp. | 2,132 |
| 93 | or/87-92 | 111,777 |
| 94 | religion/ | 63,642 |
| 95 | religi*.mp. | 79,254 |
| 96 | or/94-95 | 79,255 |
| 97 | "social determinants of health"/ | 3,426 |
| 98 | social aspect/ | 71279 |
| 99 | working poor/ | 25 |
| 100 | exp social hierarchy/ | 6,130 |
| 101 | socioeconomics/ | 130,782 |
| 102 | disparit*.mp. | 78,803 |
| 103 | inequalit*.mp. | 29,136 |
| 104 | inequit*.mp. | 8,125 |
| 105 | equity.mp. | 13,521 |
| 106 | deprivation.mp. | 95,332 |
| 107 | gini.mp. | 1,058 |
| 108 | concentration index.mp. | 1,321 |
| 109 | social welfare/ | 17,143 |
| 110 | social class/ | 30,027 |
| 111 | poverty/ | 39,507 |
| 112 | social status/ | 73,787 |
| 113 | social background/ | 450 |
| 114 | social class*.mp. | 35,043 |
| 115 | social determinants.mp. | 6,998 |
| 116 | social status.mp. | 76,926 |
| 117 | social position.mp. | 1,021 |
| 118 | social background.mp. | 1,630 |
| 119 | social circumstance*.mp. | 1,407 |
| 120 | socio-economic.mp. | 34,008 |
| 121 | socioeconomic.mp. | 87,317 |
| 122 | sociodemographic.mp. | 42,976 |
| 123 | socio-demographic.mp. | 27,196 |
| 124 | SES.mp. | 22,315 |
| 125 | disadvantaged.mp. | 12,094 |
| 126 | impoverished.mp. | 3,409 |
| 127 | poverty.mp. | 48,589 |
| 128 | economic level.mp. | 1,177 |
| 129 | assets index.mp. | 19 |
| 130 | income*.mp. | 140,750 |
| 131 | or/97-130 | 710,127 |
| 132 | exp social isolation/ | 20,351 |
| 133 | social capital/ | 1,805 |
| 134 | social stigma/ | 5,261 |

| Embase specific terms | | |
|---|---|---|
| 135 | social support/ | 76,168 |
| 136 | social environment/ | 29,908 |
| 137 | trust/ | 13,092 |
| 138 | exp social exclusion/ | 1,062 |
| 139 | anomie/ | 34 |
| 140 | social participation/ | 4,188 |
| 141 | social exclusion.mp. | 2,251 |
| 142 | (social adj (capital or cohes* or organis* or organiz*)).mp. | 6,346 |
| 143 | (community adj3 (cohes* or participa*)).mp. | 17,991 |
| 144 | ((neighbourhood or neighborhood) adj cohes*).mp. | 121 |
| 145 | social relationships.mp. | 5,541 |
| 146 | social network*.mp. | 19,683 |
| 147 | collective efficacy.mp. | 351 |
| 148 | civil society.mp. | 1,617 |
| 149 | informal social control.mp. | 96 |
| 150 | neighbo*rhood disorder.mp. | 161 |
| 151 | social disorgani?ation.mp. | 209 |
| 152 | anomie.mp. | 254 |
| 153 | social support.mp. | 85,365 |
| 154 | social participation.mp. | 5,662 |
| 155 | trust.mp. | 45,136 |
| 156 | emotional support.mp. | 6,485 |
| 157 | psychosocial support.mp. | 4,051 |
| 158 | community capital.mp. | 7 |
| 159 | neighbo*rhood cohesion.mp. | 117 |
| 160 | social influence.mp. | 1,803 |
| 161 | (soci*context* or soci*-context*).mp. | 10,198 |
| 162 | or/132-161 | 237,221 |
| 163 | health disparity/ | 13,846 |
| 164 | health equity/ | 1,005 |
| 165 | health care access/ | 50,631 |
| 166 | health*care disparit*.mp. | 907 |
| 167 | health care disparit*.mp. | 11,985 |
| 168 | health status disparit*.mp. | 351 |
| 169 | health disparit*.mp. | 20,101 |
| 170 | health inequalit*.mp. | 5,092 |
| 171 | health inequit*.mp. | 1,385 |
| 172 | medically underserved.mp. | 2,093 |
| 173 | or/163-172 | 84,892 |
| 174 | 32 or 70 or 75 or 86 or 93 or 96 or 131 or 162 or 173 | 2,233,531 |

| Embase non-specific terms | | |
|---|---|---|
| 1 | potential determinants.mp. | 1,755 |
| 2 | significant correlates of.mp. | 1,198 |
| 3 | (independent correlates or independent association*).mp. | 12,183 |
| 4 | variables associated with.mp. | 12,953 |
| 5 | determinants of.mp. | 158,678 |
| 6 | factors associated with.mp. | 127,294 |
| 7 | identif* determinants.mp. | 2,089 |
| 8 | (more likely or less likely or just as likely).mp. | 316,656 |
| 9 | risk factors for.mp. | 519,708 |

| | | |
|---|---|---|
| 10 | (significantly related to or significant predictor).mp. | 64,010 |
| 11 | (also adj2 associated with).mp. | 72,979 |
| 12 | (at increased risk or at decreased risk).mp. | 43,017 |
| 13 | association* between.mp. | 534,929 |
| 14 | (positively associated or negatively associated).mp. | 58,485 |
| 15 | differed by.mp. | 12,286 |
| 16 | (were high* amongst or were low* amongst).mp. | 1,580 |
| 17 | (inverse relationship with or inversely associated with or inversely related to).mp. | 44,456 |
| 18 | reverse association.mp. | 220 |
| 19 | differentially affects.mp. | 2,357 |
| 20 | evidence of a link between.mp. | 1,688 |
| 21 | (significantly adj3 likelihood of).mp. | 2,807 |
| 22 | protective factors for.mp. | 9,416 |
| 23 | (differ* adj2 according to).mp. | 32,849 |
| 24 | (inverse adj2 gradient).mp. | 236 |
| 25 | (positive adj2 gradient).mp. | 434 |
| 26 | (negative adj2 gradient).mp. | 344 |
| 27 | (trends were adj3 across).mp. | 1,394 |
| 28 | (related to adj3 variable*).mp. | 23,058 |
| 29 | (differences were adj3 explained by).mp. | 8,363 |
| 30 | (significant among or no# significant among).mp. | 3,724 |
| 31 | or/1-30 | 1,734,459 |

| **Embase combined strategy** | |
|---|---|
| [Specific strategy line] 174 OR [non-specific strategy line] 31 | 3,515,240 |

**MEDLINE validation set articles**


1. Walter HJ, Hofman A, Connelly PA, Barrett LT, Kost KL: Primary prevention of chronic disease in childhood: changes in risk factors after one year of intervention. American Journal of Epidemiology 1985, 122:772-781.

2. Gerald LB, Anderson A, Johnson GD, Hoff C, Trimm RF: Social class, social support and obesity risk in children. Child: Care, Health & Development 1994, 20:145-163.

3. Duran-Tauleria E, Rona RJ, Chinn S: Factors associated with weight for height and skinfold thickness in British children. Journal of Epidemiology & Community Health 1995, 49:466-473.

4. Kaplan GA, Pamuk ER, Lynch JW, Cohen RD, Balfour JL: Inequality in income and mortality in the United States: analysis of mortality and potential pathways.[Erratum appears in BMJ 1996 May 18;312(7041):1253]. BMJ 1996, 312:999-1003.

5. Kimm SY, Obarzanek E, Barton BA, Aston CE, Similo SL, Morrison JA, Sabry ZI, Schreiber GB, McMahon RP: Race, socioeconomic status, and obesity in 9- to 10-year-old girls: the NHLBI Growth and Health Study. Annals of Epidemiology 1996, 6:266-275.

6. Overstreet S, Holmes CS, Dunlap WP, Frentz J: Sociodemographic risk factors to disease control in children with diabetes. Diabetic Medicine 1997, 14:153-157.

7. Palta M, LeCaire T, Daniels K, Shen G, Allen C, D'Alessio D: Risk factors for hospitalization in a cohort with type 1 diabetes. Wisconsin Diabetes Registry. American Journal of Epidemiology 1997, 146:627-636.

8. Alaimo K, Briefel RR, Frongillo EA, Jr., Olson CM: Food insufficiency exists in the United States: results from the third National Health and Nutrition Examination Survey (NHANES III). American Journal of Public Health 1998, 88:419-426.

9. Robinson TN: Reducing children's television viewing to prevent obesity: a randomized controlled trial. JAMA 1999, 282:1561-1567.

10. Strauss RS, Knight J: Influence of the home environment on the development of obesity in children. Pediatrics 1999, 103:e85.

11. Baughcum AE, Chamberlin LA, Deeks CM, Powers SW, Whitaker RC: Maternal perceptions of overweight preschool children. Pediatrics 2000, 106:1380-1386.

12. Alaimo K, Olson CM, Frongillo EA, Jr.: Low family income and food insufficiency in relation to overweight in US children: is there a paradox? Archives of Pediatrics & Adolescent Medicine 2001, 155:1161-1167.

13. Robinson TN, Kiernan M, Matheson DM, Haydel KF: Is parental control over children's eating associated with childhood obesity? Results from a population-based sample of third graders. Obesity Research 2001, 9:306-312.

14. Selten JP, Veen N, Feller W, Blom JD, Schols D, Camoenie W, Oolders J, van der Velden M, Hoek HW, Rivero VM, et al: Incidence of psychotic disorders in immigrant groups to The Netherlands. British Journal of Psychiatry 2001, 178:367-372.

15. Van Lenthe FJ, Boreham CA, Twisk JW, Strain JJ, Savage JM, Smith GD: Socio-economic position and coronary heart disease risk factors in youth. Findings from the Young Hearts Project in Northern Ireland. European Journal of Public Health 2001, 11:43-50.

16. Wang Y: Cross-national comparison of childhood obesity: the epidemic and the relationship between obesity and socioeconomic status. International Journal of Epidemiology 2001, 30:1129-1136.

17. Bartley M, Plewis I: Accumulated labour market disadvantage and limiting long-term illness: data from the 1971-1991 Office for National Statistics' Longitudinal Study. International Journal of Epidemiology 2002, 31:336-341.

18. Hafner JW, Jr., Belknap SM, Squillante MD, Bucheit KA: Adverse drug events in emergency department patients. Annals of Emergency Medicine 2002, 39:258-267.

19. Langnase K, Mast M, Muller MJ: Social class differences in overweight of prepubertal children in northwest Germany. International Journal of Obesity & Related Metabolic Disorders: Journal of the International Association for the Study of Obesity 2002, 26:566-572.

20. Margetts BM, Mohd Yusof S, Al Dallal Z, Jackson AA: Persistence of lower birth weight in second generation South Asian babies born in the United Kingdom. Journal of Epidemiology & Community Health 2002, 56:684-687.

21. Ball K, Mishra GD, Crawford D: Social factors and obesity: an investigation of the role of health behaviours. International Journal of Obesity & Related Metabolic Disorders: Journal of the International Association for the Study of Obesity 2003, 27:394-403.

22. Charles H, Good CB, Hanusa BH, Chang CC, Whittle J: Racial differences in adherence to cardiac medications. Journal of the National Medical Association 2003, 95:17-27.

23. Gordon-Larsen P, Adair LS, Popkin BM: The relationship of ethnicity, socioeconomic factors, and overweight in US adolescents.[Erratum appears in Obes Res. 2003 Apr;11(4):597]. Obesity Research 2003, 11:121-129.

24. Haas JS, Lee LB, Kaplan CP, Sonneborn D, Phillips KA, Liang SY: The association of race, socioeconomic status, and health insurance status with the prevalence of overweight among children and adolescents. American Journal of Public Health 2003, 93:2105-2110.

25. Osler M, Andersen AM, Due P, Lund R, Damsgaard MT, Holstein BE: Socioeconomic position in early life, birth weight, childhood cognitive function, and adult mortality. A longitudinal study of Danish men born in 1953.[Erratum appears in J Epidemiol Community Health. 2003 Dec;57(12):995]. Journal of Epidemiology & Community Health 2003, 57:681-686.

26. Datar A, Sturm R: Physical education in elementary school and body mass index: evidence from the early childhood longitudinal study. American Journal of Public Health 2004, 94:1501-1506.

27. Kain J, Uauy R, Albala, Vio F, Cerda R, Leyton B: School-based obesity prevention in Chilean primary school children: methodology and evaluation of a controlled study. International Journal of Obesity & Related Metabolic Disorders: Journal of the International Association for the Study of Obesity 2004, 28:483-493.

28. Staats PS, Markowitz J, Schein J: Incidence of constipation associated with long-acting opioid therapy: a comparative study. Southern Medical Journal 2004, 97:129-134.

29. Artazcoz L, Benach J, Borrell C, Cortes I: Social inequalities in the impact of flexible employment on different domains of psychosocial health. Journal of Epidemiology & Community Health 2005, 59:761-767.

30. Gillis L, McDowell M, Bar-Or O: Relationship between summer vacation weight gain and lack of success in a pediatric weight control program. Eating Behaviors 2005, 6:137-143.

31. Hofferth SL, Curtin S: Poverty, food programs, and childhood obesity. Journal of Policy Analysis & Management 2005, 24:703-726.

32. Romon M, Duhamel A, Collinet N, Weill J: Influence of social class on time trends in BMI distribution in 5-year-old French children from 1989 to 1999. International Journal of Obesity 2005, 29:54-59.

33. Van Wijk BL, Klungel OH, Heerdink ER, de Boer A: Rate and determinants of 10-year persistence with antihypertensive drugs. Journal of Hypertension 2005, 23:2101-2107.

34. Will B, Zeeb H, Baune BT: Overweight and obesity at school entry among migrant and German children: a cross-sectional study. BMC Public Health 2005, 5:45.

35. Delva J, O'Malley PM, Johnston LD: Racial/ethnic and socioeconomic status differences in overweight and health-related behaviors among American students: national trends 1986-2003. Journal of Adolescent Health 2006, 39:536-545.

36. Gallo LC, Smith TW, Cox CM: Socioeconomic status, psychosocial processes, and perceived health: an interpersonal perspective. Annals of Behavioral Medicine 2006, 31:109-119.

37. Kramer JM, Hammill B, Anstrom KJ, Fetterolf D, Snyder R, Charde JP, Hoffman BS, Allen LaPointe N, Peterson E: National evaluation of adherence to beta-blocker therapy for 1 year after

acute myocardial infarction in patients with commercial health insurance. American Heart Journal 2006, 152:454.e451-458.

38. Ness AR, Leary S, Reilly J, Wells J, Tobias J, Clark E, Smith GD, Team AS: The social patterning of fat and lean mass in a contemporary cohort of children. International Journal of Pediatric Obesity 2006, 1:59-61.

39. Rhee KE, Lumeng JC, Appugliese DP, Kaciroti N, Bradley RH: Parenting styles and overweight status in first grade. Pediatrics 2006, 117:2047-2054.

40. Asuncion MM, Shaheen M, Ganesan K, Velasques J, Teklehaimanot S, Pan D, Norris K: Increase in hypoglycemic admissions: California hospital discharge data. Ethnicity & Disease 2007, 17:536-540.

41. Aujesky D, Long JA, Fine MJ, Ibrahim SA: African American race was associated with an increased risk of complications following venous thromboembolism. Journal of Clinical Epidemiology 2007, 60:410-416.

42. Chandola T, Ferrie J, Sacker A, Marmot M: Social inequalities in self reported health in early old age: follow-up of prospective cohort study. BMJ 2007, 334:990.

43. Delva J, Johnston LD, O'Malley PM: The epidemiology of overweight and related lifestyle behaviors: racial/ethnic and socioeconomic status differences among American youth. American Journal of Preventive Medicine 2007, 33:S178-186.

44. Emerson E, Hatton C: Poverty, socio-economic position, social capital and the health of children and adolescents with intellectual disabilities in Britain: a replication. Journal of Intellectual Disability Research 2007, 51:866-874.

45. Gudmundsson J, Sulem P, Steinthorsdottir V, Bergthorsson JT, Thorleifsson G, Manolescu A, Rafnar T, Gudbjartsson D, Agnarsson BA, Baker A, et al: Two variants on chromosome 17 confer prostate cancer risk, and the one in TCF2 protects against type 2 diabetes. Nature Genetics 2007, 39:977-983.

46. Hernandez-Valero MA, Wilkinson AV, Forman MR, Etzel CJ, Cao Y, Barcenas CH, Strom SS, Spitz MR, Bondy ML: Maternal BMI and country of birth as indicators of childhood obesity in children of Mexican origin. Obesity 2007, 15:2512-2519.

47. Panico L, Bartley M, Marmot M, Nazroo JY, Sacker A, Kelly YJ: Ethnic variation in childhood asthma and wheezing illnesses: findings from the Millennium Cohort Study. International Journal of Epidemiology 2007, 36:1093-1102.

48. Rasmussen JN, Gislason GH, Rasmussen S, Abildstrom SZ, Schramm TK, Kober L, Diderichsen F, Osler M, Torp-Pedersen C, Madsen M: Use of statins and beta-blockers after acute myocardial infarction according to income and education. Journal of Epidemiology & Community Health 2007, 61:1091-1097.

49. Taira DA, Gelber RP, Davis J, Gronley K, Chung RS, Seto TB: Antihypertensive adherence and drug class among Asian Pacific Americans. Ethnicity & Health 2007, 12:265-281.

50. von Hippel PT, Powell B, Downey DB, Rowland NJ: The effect of school on overweight in childhood: gain in body mass index during the school year and during summer vacation. American Journal of Public Health 2007, 97:696-702.

51. Brixner DI, Jackson KC, 2nd, Sheng X, Nelson RE, Keskinaslan A: Assessment of adherence, persistence, and costs among valsartan and hydrochlorothiazide retrospective cohorts in free-and fixed-dose combinations. Current Medical Research & Opinion 2008, 24:2597-2607.

52. Engstrom K, Mattsson F, Jarleborg A, Hallqvist J: Contextual social capital as a risk factor for poor self-rated health: a multilevel analysis. Social Science & Medicine 2008, 66:2268-2280.

53. Gray L, Leyland AH: Overweight status and psychological well-being in adolescent boys and girls: a multilevel analysis. European Journal of Public Health 2008, 18:616-621.

54. Hall JV, Brajer V, Lurmann FW: Measuring the gains from improved air quality in the San Joaquin Valley. Journal of Environmental Management 2008, 88:1003-1015.

55. Lutfiyya MN, Garcia R, Dankwa CM, Young T, Lipsky MS: Overweight and obese prevalence rates in African American and Hispanic children: an analysis of data from the 2003-2004 National Survey of Children's Health. Journal of the American Board of Family Medicine: JABFM 2008, 21:191-199.

56. Mikolajczyk RT, Richter M: Associations of behavioural, psychosocial and socioeconomic factors with over- and underweight among German adolescents. International Journal of Public Health 2008, 53:214-220.

57. Mitchell R, Popham F: Effect of exposure to natural environment on health inequalities: an observational population study. Lancet 2008, 372:1655-1660.

58. Patel BV, Remigio-Baker RA, Thiebaud P, Preblick R, Plauschinat C: Improved persistence and adherence to diuretic fixed-dose combination therapy compared to diuretic monotherapy. BMC Family Practice 2008, 9:61.

59. Richmond TK, Subramanian SV: School level contextual factors are associated with the weight status of adolescent males and females. Obesity 2008, 16:1324-1330.

60. Simon C, Schweitzer B, Oujaa M, Wagner A, Arveiler D, Triby E, Copin N, Blanc S, Platat C: Successful overweight prevention in adolescents by increasing physical activity: a 4-year randomized controlled intervention.[Erratum appears in Int J Obes (Lond). 2008 Oct;32(10):1606]. International Journal of Obesity 2008, 32:1489-1498.

61. Singh GK, Kogan MD, van Dyck PC: A multilevel analysis of state and regional disparities in childhood and adolescent obesity in the United States. Journal of Community Health 2008, 33:90-102.

62. Stafford M, Gimeno D, Marmot MG: Neighbourhood characteristics and trajectories of health functioning: a multilevel prospective analysis. European Journal of Public Health 2008, 18:604-610.

63. Zandieh SO, Goldmann DA, Keohane CA, Yoon C, Bates DW, Kaushal R: Risk factors in preventable adverse drug events in pediatric outpatients. Journal of Pediatrics 2008, 152:225-231.

64. Bae HJ, Park J: Health benefits of improving air quality in the rapidly aging Korean society. Science of the Total Environment 2009, 407:5971-5977.

65. Becares L, Nazroo J, Stafford M: The buffering effects of ethnic density on experienced racism and health. Health & Place 2009, 15:670-678.

66. Brophy S, Cooksey R, Gravenor MB, Mistry R, Thomas N, Lyons RA, Williams R: Risk factors for childhood obesity at age 5: analysis of the millennium cohort study. BMC Public Health 2009, 9:467.

67. Gater R, Tomenson B, Percival C, Chaudhry N, Waheed W, Dunn G, Macfarlane G, Creed F: Persistent depressive disorders and social stress in people of Pakistani origin and white Europeans in UK. Social Psychiatry & Psychiatric Epidemiology 2009, 44:198-207.

68. Hawkins SS, Cole TJ, Law C, Millennium Cohort Study Child Health G: An ecological systems approach to examining risk factors for early childhood overweight: findings from the UK Millennium Cohort Study. Journal of Epidemiology & Community Health 2009, 63:147-155.

69. Kelly Y, Panico L, Bartley M, Marmot M, Nazroo J, Sacker A: Why does birthweight vary among ethnic groups in the UK? Findings from the Millennium Cohort Study. Journal of Public Health 2009, 31:131-137.

70. Maas J, Verheij RA, de Vries S, Spreeuwenberg P, Schellevis FG, Groenewegen PP: Morbidity is related to a green living environment. Journal of Epidemiology & Community Health 2009, 63:967-973.

71. Perez L, Sunyer J, Kunzli N: Estimating the health and economic benefits associated with reducing air pollution in the Barcelona metropolitan area (Spain). Gaceta Sanitaria 2009, 23:287-294.

72. Sung SK, Lee SG, Lee KS, Kim DS, Kim KH, Kim KY: First-year treatment adherence among outpatients initiating antihypertensive medication in Korea: results of a retrospective claims review. Clinical Therapeutics 2009, 31:1309-1320.

73. Voorhees CC, Catellier DJ, Ashwood JS, Cohen DA, Rung A, Lytle L, Conway TL, Dowda M: Neighborhood socioeconomic status and non school physical activity and body mass index in adolescent girls. Journal of Physical Activity & Health 2009, 6:731-740.

74. Wake M, Baur LA, Gerner B, Gibbons K, Gold L, Gunn J, Levickis P, McCallum Z, Naughton G, Sanci L, Ukoumunne OC: Outcomes and costs of primary care surveillance and intervention for overweight or obese children: the LEAP 2 randomised controlled trial. BMJ 2009, 339:b3308.

75. Waters KM, Le Marchand L, Kolonel LN, Monroe KR, Stram DO, Henderson BE, Haiman CA: Generalizability of associations from prostate cancer genome-wide association studies in multiple populations. Cancer Epidemiology, Biomarkers & Prevention 2009, 18:1285-1289.

76. Wong MC, Jiang JY, Griffiths SM: Factors associated with compliance, discontinuation and switching of calcium channel blockers in 20,156 Chinese patients. American Journal of Hypertension 2009, 22:904-910.

77. Abdou CM, Dunkel Schetter C, Campos B, Hilmert CJ, Dominguez TP, Hobel CJ, Glynn LM, Sandman C: Communalism predicts prenatal affect, stress, and physiology better than ethnicity and socioeconomic status. Cultural Diversity & Ethnic Minority Psychology 2010, 16:395-403.

78. Anderson SE, Whitaker RC: Household routines and obesity in US preschool-aged children. Pediatrics 2010, 125:420-428.

79. Black MM, Hager ER, Le K, Anliker J, Arteaga SS, Diclemente C, Gittelsohn J, Magder L, Papas M, Snitker S, et al: Challenge! Health promotion/obesity prevention mentorship model among urban, black adolescents. Pediatrics 2010, 126:280-288.

80. Bourgeois FT, Shannon MW, Valim C, Mandl KD: Adverse drug events in the outpatient setting: an 11-year national analysis. Pharmacoepidemiology & Drug Safety 2010, 19:901-910.

81. Flegal KM, Carroll MD, Ogden CL, Curtin LR: Prevalence and trends in obesity among US adults, 1999-2008. JAMA 2010, 303:235-241.

82. Friedman O, McAlister FA, Yun L, Campbell NR, Tu K, Canadian Hypertension Education Program Outcomes Research T: Antihypertensive drug persistence and compliance among newly treated elderly hypertensives in ontario. American Journal of Medicine 2010, 123:173-181.

83. Glader EL, Sjolander M, Eriksson M, Lundberg M: Persistent use of secondary preventive drugs declines rapidly during the first 2 years after stroke. Stroke 2010, 41:397-401.

84. Howe LD, Galobardes B, Sattar N, Hingorani AD, Deanfield J, Ness AR, Davey-Smith G, Lawlor DA: Are there socioeconomic inequalities in cardiovascular risk factors in childhood, and are they mediated by adiposity? Findings from a prospective cohort study. International Journal of Obesity 2010, 34:1149-1159.

85. Ibuka Y, Chapman GB, Meyers LA, Li M, Galvani AP: The dynamics of risk perceptions and precautionary behavior in response to 2009 (H1N1) pandemic influenza. BMC Infectious Diseases 2010, 10:296.

86. Lau JT, Yeung NC, Choi KC, Cheng MY, Tsui HY, Griffiths S: Factors in association with acceptability of A/H1N1 vaccination during the influenza A/H1N1 pandemic phase in the Hong Kong general population. Vaccine 2010, 28:4632-4637.

87. Li J, Hooker NH: Childhood obesity and schools: evidence from the national survey of children's health. Journal of School Health 2010, 80:96-103.

88. Liao Q, Cowling B, Lam WT, Ng MW, Fielding R: Situational awareness and health protective responses to pandemic influenza A (H1N1) in Hong Kong: a cross-sectional study. PLoS ONE [Electronic Resource] 2010, 5:e13350.

89. Park JH, Cheong HK, Son DY, Kim SU, Ha CM: Perceptions and behaviors related to hand hygiene for the prevention of H1N1 influenza transmission among Korean university students during the peak pandemic period. BMC Infectious Diseases 2010, 10:222.

90. Schwarzinger M, Flicoteaux R, Cortarenoda S, Obadia Y, Moatti JP: Low acceptability of A/H1N1 pandemic vaccination in French adult population: did public health policy fuel public dissonance? PLoS ONE [Electronic Resource] 2010, 5:e10199.

91. Singh GK, Siahpush M, Kogan MD: Rising social inequalities in US childhood obesity, 2003-2007.[Erratum appears in Ann Epidemiol. 2010 Mar;20(3):250]. Annals of Epidemiology 2010, 20:40-52.

92. Stamatakis E, Wardle J, Cole TJ: Childhood obesity and overweight prevalence trends in England: evidence for growing socioeconomic disparities. International Journal of Obesity 2010, 34:41-47.

93. Stamatakis E, Zaninotto P, Falaschetti E, Mindell J, Head J: Time trends in childhood and adolescent obesity in England from 1995 to 2007 and projections of prevalence to 2015. Journal of Epidemiology & Community Health 2010, 64:167-174.

94. Wong LP, Sam IC: Factors influencing the uptake of 2009 H1N1 influenza vaccine in a multiethnic Asian population. Vaccine 2010, 28:4499-4505.

95. Wong LP, Sam IC: Public sources of information and information needs for pandemic influenza A(H1N1). Journal of Community Health 2010, 35:676-682.

96. Wong MC, Jiang JY, Griffiths SM: Factors associated with antihypertensive drug compliance in 83,884 Chinese patients: a cohort study. Journal of Epidemiology & Community Health 2010, 64:895-901.

97. Zeng F, Patel BV, Andrews L, Frech-Tamas F, Rudolph AE: Adherence and persistence of single-pill ARB/CCB combination therapy compared to multiple-pill ARB/CCB regimens. Current Medical Research & Opinion 2010, 26:2877-2887.

98. Aida J, Kondo K, Kondo N, Watt RG, Sheiham A, Tsakos G: Income inequality, social capital and self-rated health and dental status in older Japanese. Social Science & Medicine 2011, 73:1561-1568.

99. Chanel O, Luchini S, Massoni S, Vergnaud JC: Impact of information on intentions to vaccinate in a potential epidemic: Swine-origin Influenza A (H1N1). Social Science & Medicine 2011, 72:142-148.

100. de Meij JS, Chinapaw MJ, van Stralen MM, van der Wal MF, van Dieren L, van Mechelen W: Effectiveness of JUMP-in, a Dutch primary school-based community intervention aimed at the promotion of physical activity. British Journal of Sports Medicine 2011, 45:1052-1057.

101. Ferrante G, Baldissera S, Moghadam PF, Carrozzi G, Trinito MO, Salmaso S: Surveillance of perceptions, knowledge, attitudes and behaviors of the Italian adult population (18-69 years) during the 2009-2010 A/H1N1 influenza pandemic. European Journal of Epidemiology 2011, 26:211-219.

102. Hamad R, Fernald L, Karlan DS: Health education for microcredit clients in Peru: a randomized controlled trial. BMC Public Health 2011, 11:51.

103. Howe LD, Tilling K, Galobardes B, Smith GD, Ness AR, Lawlor DA: Socioeconomic disparities in trajectories of adiposity across childhood. International Journal of Pediatric Obesity 2011, 6:e144-153.

104. Huang C, Soldo BJ, Elo IT: Do early-life conditions predict functional health status in adulthood? The case of Mexico. Social Science & Medicine 2011, 72:100-107.

105. Jehu-Appiah C, Aryeetey G, Spaan E, de Hoop T, Agyepong I, Baltussen R: Equity aspects of the National Health Insurance Scheme in Ghana: Who is enrolling, who is not and why? Social Science & Medicine 2011, 72:157-165.

106. Kavanagh AM, Bentley RJ, Mason KE, McVernon J, Petrony S, Fielding J, LaMontagne AD, Studdert DM: Sources, perceived usefulness and understanding of information disseminated to families who entered home quarantine during the H1N1 pandemic in Victoria, Australia: a cross-sectional study. BMC Infectious Diseases 2011, 11:2.

107. Khullar D, Oreskovic NM, Perrin JM, Goodman E: Optimism and the socioeconomic status gradient in adolescent adiposity. Journal of Adolescent Health 2011, 49:553-555.

108. Lin Y, Huang L, Nie S, Liu Z, Yu H, Yan W, Xu Y: Knowledge, attitudes and practices (KAP) related to the pandemic (H1N1) 2009 among Chinese general population: a telephone survey. BMC Infectious Diseases 2011, 11:128.

109. Myers LB, Goodwin R: Determinants of adults' intention to vaccinate against pandemic swine flu. BMC Public Health 2011, 11:15.

110. Nemet D, Geva D, Eliakim A: Health promotion intervention in low socioeconomic kindergarten children. Journal of Pediatrics 2011, 158:796-801.e791.

111. Plough A, Bristow B, Fielding J, Caldwell S, Khan S: Pandemics and health equity: lessons learned from the H1N1 response in Los Angeles County. Journal of Public Health Management & Practice 2011, 17:20-27.

112. Son M, Kim J, Oh J, Kawachi I: Inequalities in childhood cancer mortality according to parental socioeconomic position: A birth cohort study in South Korea. Social Science & Medicine 2011, 72:108-115.

113. Taveras EM, Gortmaker SL, Hohman KH, Horan CM, Kleinman KP, Mitchell K, Price S, Prosser LA, Rifas-Shiman SL, Gillman MW: Randomized controlled trial to improve primary care to prevent and manage childhood obesity: the High Five for Kids study. Archives of Pediatrics & Adolescent Medicine 2011, 165:714-722.

114. Wang W, Sulzbach S, De S: Utilization of HIV-related services from the private health sector: A multi-country analysis. Social Science & Medicine 2011, 72:216-223.

115. Wong MC, Jiang JY, Griffiths SM: Factors associated with compliance to thiazide diuretics among 8551 Chinese patients. Journal of Clinical Pharmacy & Therapeutics 2011, 36:179-186.

116. Zhang J, Himes JH, Hannan PJ, Arcan C, Smyth M, Rock BH, Story M: Summer effects on body mass index (BMI) gain and growth patterns of American Indian children from kindergarten to first grade: a prospective study. BMC Public Health 2011, 11:951.

117. Burgi F, Niederer I, Schindler C, Bodenmann P, Marques-Vidal P, Kriemler S, Puder JJ: Effect of a lifestyle intervention on adiposity and fitness in socially disadvantaged subgroups of preschoolers: a cluster-randomized trial (Ballabeina). Preventive Medicine 2012, 54:335-340.

118. Cesaroni G, Boogaard H, Jonkers S, Porta D, Badaloni C, Cattani G, Forastiere F, Hoek G: Health benefits of traffic-related air pollution reduction in different socioeconomic groups: the effect of low-emission zoning in Rome. Occupational & Environmental Medicine 2012, 69:133-139.

119. Evans CD, Eurich DT, Remillard AJ, Shevchuk YM, Blackburn D: First-fill medication discontinuations and nonadherence to antihypertensive therapy: an observational study. American Journal of Hypertension 2012, 25:195-203.

120. Gaygisiz U, Gaygisiz E, Ozkan T, Lajunen T: Individual differences in behavioral reactions to H1N1 during a later stage of the epidemic. Journal of Infection and Public Health 2012, 5:9-21.

121. Gopinath B, Baur LA, Burlutsky G, Robaei D, Mitchell P: Socio-economic, familial and perinatal factors associated with obesity in Sydney schoolchildren. Journal of Paediatrics & Child Health 2012, 48:44-51.

122. Jimenez N, Anderson GD, Shen DD, Nielsen SS, Farin FM, Seidel K, Lynn AM: Is ethnicity associated with morphine's side effects in children? Morphine pharmacokinetics, analgesic response, and side effects in children having tonsillectomy. Paediatric Anaesthesia 2012, 22:669-675.

123. Mak KK, Lai CM: Knowledge, risk perceptions, and preventive precautions among Hong Kong students during the 2009 influenza A (H1N1) pandemic. American Journal of Infection Control 2012, 40:273-275.

124. Moffett BS, Ung M, Bomgaars L: Risk factors for elevated INR values during warfarin therapy in hospitalized pediatric patients. Pediatric Blood & Cancer 2012, 58:941-944.

125. Rossen LM, Schoendorf KC: Measuring health disparities: trends in racial-ethnic and socioeconomic disparities in obesity among 2- to 18-year old youth in the United States, 2001-2010. Annals of Epidemiology 2012, 22:698-704.

126. Sadhasivam S, Chidambaran V, Ngamprasertwong P, Esslinger HR, Prows C, Zhang X, Martin LJ, McAuliffe J: Race and unequal burden of perioperative pain and opioid related adverse effects in children. Pediatrics 2012, 129:832-838.

127. Taylor AW, Winefield H, Kettler L, Roberts R, Gill TK: A population study of 5 to 15 year olds: full time maternal employment not associated with high BMI. The importance of screen-based activity, reading for pleasure and sleep duration in children's BMI. Maternal & Child Health Journal 2012, 16:587-599.

128. Trippe BS, Shepherd MD, Coulter FC, Bhargava A, Brett J, Chu PL, Oyer DS: Efficacy and safety of biphasic insulin aspart 70/30 in type 2 diabetes patients of different race or ethnicity (INITIATEplus trial). Current Medical Research & Opinion 2012, 28:1203-1211.

129. Walter D, Bohmer M, Reiter S, Krause G, Wichmann O: Risk perception and information-seeking behaviour during the 2009/10 influenza A(H1N1)pdm09 pandemic in Germany. Euro Surveillance: Bulletin Europeen sur les Maladies Transmissibles = European Communicable Disease Bulletin 2012, 17:29.

130. Wong MC, Lau RK, Jiang JY, Griffiths SM: Discontinuation of angiotensin-converting enzyme inhibitors: a cohort study. Journal of Clinical Pharmacy & Therapeutics 2012, 37:335-341.

131. Aitsi-Selmi A, Chen R, Shipley MJ, Marmot MG: Education is associated with lower levels of abdominal obesity in women with a non-agricultural occupation: an interaction study using China's Four Provinces survey. BMC Public Health 2013, 13:769.

132. Brooks-Gunn J, Schneider W, Waldfogel J: The Great Recession and the risk for child maltreatment. Child Abuse & Neglect 2013, 37:721-729.

133. Jansen PW, Mensah FK, Nicholson JM, Wake M: Family and neighbourhood socioeconomic inequalities in childhood trajectories of BMI and overweight: longitudinal study of Australian children.[Erratum appears in PLoS One. 2013;8(7). doi:10.1371/annotation/f7e5e1f3-77f6-4c56-b0ba-53b54a86df14]. PLoS ONE [Electronic Resource] 2013, 8:e69676.

134. Kessler ER, Shah M, Gruschkus SK, Raju A: Cost and quality implications of opioid-based postsurgical pain control using administrative claims data from a large health system: opioid-related adverse events and their impact on clinical and economic outcomes. Pharmacotherapy:The Journal of Human Pharmacology & Drug Therapy 2013, 33:383-391.

135. Moreno JP, Johnston CA, Woehler D: Changes in weight over the school year and summer vacation: results of a 5-year longitudinal study. Journal of School Health 2013, 83:473-477.

136. Pardo-Crespo MR, Narla NP, Williams AR, Beebe TJ, Sloan J, Yawn BP, Wheeler PH, Juhn YJ: Comparison of individual-level versus area-level socioeconomic measures in assessing health outcomes of children in Olmsted County, Minnesota. Journal of Epidemiology & Community Health 2013, 67:305-310.

137. Schwanke Khilji SU, Rudge JW, Drake T, Chavez I, Borin K, Touch S, Coker R, CamFlu Project C: Distribution of selected healthcare resources for influenza pandemic response in Cambodia. International Journal for Equity in Health 2013, 12:82.

138. Sheridan E, Wright J, Small N, Corry PC, Oddie S, Whibley C, Petherick ES, Malik T, Pawson N, McKinney PA, Parslow RC: Risk factors for congenital anomaly in a multiethnic birth cohort: an analysis of the Born in Bradford study. Lancet 2013, 382:1350-1359.

139. Thibault H, Carriere C, Langevin C, Kossi Deti E, Barberger-Gateau P, Maurice S: Prevalence and factors associated with overweight and obesity in French primary-school children. Public Health Nutrition 2013, 16:193-201.

140. Zhao Y, You J, Wright J, Guthridge SL, Lee AH: Health inequity in the Northern Territory, Australia. International Journal for Equity in Health 2013, 12:79.

141. Baranowski T, O'Connor T, Johnston C, Hughes S, Moreno J, Chen TA, Meltzer L, Baranowski J: School year versus summer differences in child weight gain: a narrative review. Childhood Obesity 2014, 10:18-24.

142. Beck AN, Finch BK, Lin SF, Hummer RA, Masters RK: Racial disparities in self-rated health: trends, explanatory factors, and the changing role of socio-demographics. Social Science & Medicine 2014, 104:163-177.

143. Branco FL, Pereira TM, Delfino BM, Brana AM, Oliart-Guzman H, Mantovani SA, Martins AC, Oliveira CS, Ramalho AA, Codeco CT, da Silva-Nunes M: Socioeconomic inequalities are still a barrier to full child vaccine coverage in the Brazilian Amazon: a cross-sectional study in Assis Brasil, Acre, Brazil. International Journal for Equity in Health 2014, 13:118.

144. Dolan P, Rudisill C: The effect of financial incentives on chlamydia testing rates: evidence from a randomized experiment. Social Science & Medicine 2014, 105:140-148.

145. Grytten J, Skau I, Sorensen RJ: Educated mothers, healthy infants. The impact of a school reform on the birth weight of Norwegian infants 1967-2005. Social Science & Medicine 2014, 105:84-92.

146. Hajizadeh M, Alam N, Nandi A: Social inequalities in the utilization of maternal care in Bangladesh: Have they widened or narrowed in recent years? International Journal for Equity in Health 2014, 13:120.

147. Schmidt NM, Lincoln AK, Nguyen QC, Acevedo-Garcia D, Osypuk TL: Examining mediators of housing mobility on adolescent asthma: results from a housing voucher experiment. Social Science & Medicine 2014, 107:136-144.

148. von Hippel PT, Lynch JL: Why are educated adults slim-Causation or selection? Social Science & Medicine 2014, 105:131-139.

149. Wang B, Deveaux L, Li X, Marshall S, Chen X, Stanton B: The impact of youth, family, peer and neighborhood risk factors on developmental trajectories of risk involvement from early through middle adolescence. Social Science & Medicine 2014, 106:43-52.

150. Ki M, Lee YH, Kim YS, Shin JY, Lim J, Nazroo J: Socioeconomic inequalities in health in the context of multimorbidity: A Korean panel study. PLoS ONE [Electronic Resource] 2017, 12:e0173770.

**Embase validation set articles**

[1-150]

1. Gerald LB, Anderson A, Johnson GD, Hoff C, Trimm RF: Social class, social support and obesity risk in children. Child: care, health and development 1994, 20:145-163.

2. Duran-Tauleria E, Rona RJ, Chinn S: Factors associated with weight for height and skinfold thickness in British children. Journal of Epidemiology and Community Health 1995, 49:466-473.

3. Bailey JE, Lee MD, Somes GW, Graham RL: Risk factors for antihypertensive medication refill failure by patients under Medicaid managed care. Clinical Therapeutics 1996, 18:1252-1262.

4. Kimm SYS, Obarzanek E, Barton BA, Aston CE, Similo SL, Morrison JA, Sabry ZI, Schreiber GB, McMahon RP: Race, socioeconomic status, and obesity in 9- to 10-year-old girls: The NHLBI growth and health study. Annals of Epidemiology 1996, 6:266-275.

5. Overstreet S, Holmes CS, Dunlap WP, Frentz J: Sociodemographic risk factors to disease control in children with diabetes. Diabetic Medicine 1997, 14:153-157.

6. Crooks DL: Child growth and nutritional status in a high-poverty community in Eastern Kentucky. American Journal of Physical Anthropology 1999, 109:129-142.

7. Goodman E: The role of socioeconomic status gradients in explaining differences in US adolescents' health. American Journal of Public Health 1999, 89:1522-1528.

8. Kromeyer-Hauschild K, Zellner K, Jaeger U, Hoyer H: Prevalence of overweight and obesity among school children in Jena (Germany). International Journal of Obesity 1999, 23:1143-1150.

9. Strauss RS, Knight J: Influence of the home environment on the development of obesity in children. Pediatrics 1999, 103:e85.

10. Baughcum AE, Chamberlin LA, Deeks CM, Powers SW, Whitaker RC: Maternal perceptions of overweight preschool children. Pediatrics 2000, 106:1380-1386.

11. Alaimo K, Olson CM, Frongillo EA, Jr.: Low family income and food insufficiency in relation to overweight in US children: Is there a Paradox? Archives of Pediatrics and Adolescent Medicine 2001, 155:1161-1167.

12. Van Lenthe FJ, Boreham CA, Twisk JWR, Strain JJ, Savage JM, Smith GD: Socio-economic position and coronary heart disease risk factors in youth: Findings from the young hearts project in Northern Ireland. European Journal of Public Health 2001, 11:43-50.

13. Aveyard P, Cheng KK, Manaseki S, Gardosi J: The risk of preterm delivery in women from different ethnic groups. BJOG: An International Journal of Obstetrics and Gynaecology 2002, 109:894-899.

14. Clancy L, Goodman P, Sinclair H, Dockery DW: Effect of air-pollution control on death rates in Dublin, Ireland: An intervention study. Lancet 2002, 360:1210-1214.

15. Hafner Jr JW, Belknap SM, Squillante MD, Bucheit KA: Adverse drug events in emergency department patients. Annals of Emergency Medicine 2002, 39:258-267.

16. Neumark-Sztainer D, Story M, Hannan PJ, Croll J: Overweight status and eating patterns among adolescents: Where do youths stand in comparison with the Healthy People 2010 objectives? American Journal of Public Health 2002, 92:844-851.

17. Cepeda MS, Farrar JT, Baumgarten M, Boston R, Carr DB, Strom BL: Side effects of opioids during short-term administration: Effect of age, gender, and race. Clinical Pharmacology and Therapeutics 2003, 74:102-112.

18. Charles H, Good CB, Hanusa BH, Chang CCH, Whittle J: Racial differences in adherence to cardiac medications. Journal of the National Medical Association 2003, 95:17-27.

19. Goodman E, Adler NE, Daniels SR, Morrison JA, Slap GB, Dolan LM: Impact of objective and subjective social status on obesity in a biracial cohort of adolescents. Obesity Research 2003, 11:1018-1026.

20. Goodman E, Slap GB, Huang B: The Public Health Impact of Socioeconomic Status on Adolescent Depression and Obesity. American Journal of Public Health 2003, 93:1844-1850.

21. Haas JS, Lee LB, Kaplan CP, Sonneborn D, Phillips KA, Liang SY: The Association of Race, Socioeconomic Status, and Health Insurance Status with the Prevalence of Overweight among Children and Adolescents. American Journal of Public Health 2003, 93:2105-2110.

22. Klein-Platat C, Wagner A, Haan MC, Arveiler D, Schlienger JL, Simon C: Prevalence and sociodemographic determinants of overweight in young French adolescents. Diabetes/Metabolism Research and Reviews 2003, 19:153-158.

23. Osler M, Andersen AMN, Due P, Lund R, Damsgaard MT, Holstein BE: Socioeconomic position in early life, birth weight, childhood cognitive function, and adult mortality. A longitudinal study of Danish men born in 1953. Journal of Epidemiology and Community Health 2003, 57:681-686.

24. Robinson TN, Killen JD, Kraemer HC, Wilson DM, Matheson DM, Haskell WL, Pruitt LA, Powell TM, Owens AS, Thompson NS, et al: Dance and reducing television viewing to prevent weight gain in African-American girls: The Stanford GEMS pilot study. Ethnicity and Disease 2003, 13:S1-65-S61-77.

25. Kain J, Uauy R, Albala, Vio F, Cerda R, Leyton B: School-based obesity prevention in Chilean primary school children: Methodology and evaluation of a controlled study. International Journal of Obesity 2004, 28:483-493.

26. Mindell J, Joffe M: Predicted health impacts of urban air quality management. Journal of Epidemiology and Community Health 2004, 58:103-113.

27. Saxena S, Ambler G, Cole TJ, Majeed A: Ethnic group differences in overweight and obese children and young people in England: Cross sectional survey. Archives of Disease in Childhood 2004, 89:30-36.

28. Gillis L, McDowell M, Bar-Or O: Relationship between summer vacation weight gain and lack of success in a pediatric weight control program. Eating Behaviors 2005, 6:137-143.

29. Will B, Zeeb H, Baune BT: Overweight and obesity at school entry among migrant and German children: A cross-sectional study. BMC Public Health 2005, 5 (no pagination).

30. Elizabeth Jesse D, Graham M, Swanson M: Psychosocial and spiritual factors associated with smoking and substance use during pregnancy in African American and White low-income women. Journal of obstetric, gynecologic, and neonatal nursing : JOGNN / NAACOG 2006, 35:68-77.

31. Gallo LC, Smith TW, Cox CM: Socioeconomic status, psychosocial processes, and perceived health: An interpersonal perspective. Annals of Behavioral Medicine 2006, 31:109-119.

32. Kelly YJ, Watt RG, Nazroo JY: Racial/ethnic differences in breastfeeding initiation and continuation in the United Kingdom and comparison with findings in the United States. Pediatrics 2006, 118:e1428-e1435.

33. Kramer JM, Hammill B, Anstrom KJ, Fetterolf D, Snyder R, Charde JP, Hoffman BS, LaPointe NA, Peterson E: National evaluation of adherence to beta-blocker therapy for 1 year after acute myocardial infarction in patients with commercial health insurance. American Heart Journal 2006, 152:454-460.

34. Rose D, Bodor JN: Household food insecurity and overweight status in young school children: Results from the early childhood longitudinal study. Pediatrics 2006, 117:464-473.

35. Turrell G, Kavanagh A, Subramanian SV: Area variation in mortality in Tasmania (Australia): The contributions of socioeconomic disadvantage, social capital and geographic remoteness. Health and Place 2006, 12:291-305.

36. Wang Y, Zhang Q: Are American children and adolescents of low socioeconomic status at increased risk of obesity? Changes in the association between overweight and family income between 1971 and 2002. American Journal of Clinical Nutrition 2006, 84:707-716.

37. Aujesky D, Long JA, Fine MJ, Ibrahim SA: African American race was associated with an increased risk of complications following venous thromboembolism. Journal of Clinical Epidemiology 2007, 60:410-416.

38. Chandola T, Ferrie J, Sacker A, Marmot M: Social inequalities in self reported health in early old age: Follow-up of prospective cohort study. British Medical Journal 2007, 334:990-993.

39. Emerson E, Hatton C: Poverty, socio-economic position, social capital and the health of children and adolescents with intellectual disabilities in Britain: A replication. Journal of Intellectual Disability Research 2007, 51:866-874.

40. Hesketh K, Crawford D, Salmon J, Jackson M, Campbell K: Associations between family circumstance and weight status of Australian children. International Journal of Pediatric Obesity 2007, 2:86-96.

41. Kalavainen MP, Korppi MO, Nuutinen OM: Clinical efficacy of group-based treatment for childhood obesity compared with routinely given individual counseling. International Journal of Obesity 2007, 31:1500-1508.

42. Panico L, Bartley M, Marmot M, Nazroo J, Sacker A, Kelly YJ: Ethnic variation in childhood asthma and wheezing illnesses: Findings from the Millennium Cohort Study. International Journal of Epidemiology 2007, 36:1093-1102.

43. Rasmussen JN, Gislason GH, Rasmussen S, Abildstrom SZ, Schramm TK, Kober L, Diderichsen F, Osler M, Torp-Pedersen C, Madsen M: Use of statins and beta-blockers after acute myocardial infarction according to income and education. Journal of Epidemiology and Community Health 2007, 61:1091-1097.

44. Taira DA, Gelber RP, Davis J, Gronley K, Chung RS, Seto TB: Antihypertensive adherence and drug class among Asian Pacific Americans. Ethnicity and Health 2007, 12:265-281.

45. Van Dijk L, Heerdink ER, Somai D, Van Dulmen S, Sluijs EM, De Ridder DT, Griens AMGF, Bensing JM: Patient risk profiles and practice variation in nonadherence to antidepressants, antihypertensives and oral hypoglycemics. BMC Health Services Research 2007, 7 (no pagination).

46. Wake M, Hardy P, Canterford L, Sawyer M, Carlin JB: Overweight, obesity and girth of Australian preschoolers: Prevalence and socio-economic correlates. International Journal of Obesity 2007, 31:1044-1051.

47. Apfelbacher CJ, Loerbroks A, Cairns J, Behrendt H, Ring J, Kramer U: Predictors of overweight and obesity in five to seven-year-old children in Germany: Results from cross-sectional studies. BMC Public Health 2008, 8 (no pagination).

48. Engstrom K, Mattsson F, Jarleborg A, Hallqvist J: Contextual social capital as a risk factor for poor self-rated health: A multilevel analysis. Social Science and Medicine 2008, 66:2268-2280.

49. Gray L, Leyland AH: Overweight status and psychological well-being in adolescent boys and girls: A multilevel analysis. European Journal of Public Health 2008, 18:616-621.

50. Mitchell R, Popham F: Effect of exposure to natural environment on health inequalities: an observational population study. The Lancet 2008, 372:1655-1660.

51. Nawal Lutfiyya M, Garcia R, Dankwa CM, Young T, Lipsky MS: Overweight and obese prevalence rates in African American and Hispanic children: An analysis of data from the 2003-2004 National Survey of Children's Health. Journal of the American Board of Family Medicine 2008, 21:191-199.

52. Richmond TK, Subramanian SV: School level contextual factors are associated with the weight status of adolescent males and females. Obesity 2008, 16:1324-1330.

53. Simon C, Schweitzer B, Oujaa M, Wagner A, Arveiler D, Triby E, Copin N, Blanc S, Platat C: Successful overweight prevention in adolescents by increasing physical activity: A 4-year randomized controlled intervention. International Journal of Obesity 2008, 32:1489-1498.

54. Singh GK, Kogan MD, Van Dyck PC: A multilevel analysis of state and regional disparities in childhood and adolescent obesity in the United States. Journal of Community Health 2008, 33:90-102.

55. Stafford M, Gimeno D, Marmot MG: Neighbourhood characteristics and trajectories of health functioning: A multilevel prospective analysis. European Journal of Public Health 2008, 18:604-610.

56. Sun J, Purcell L, Gao Z, Isaacs SD, Wiley KE, Hsu FC, Liu W, Duggan D, Carpten JD, Gronberg H, et al: Association between sequence variants at 17q12 and 17q24.3 and prostate cancer risk in European and African Americans. Prostate 2008, 68:691-697.

57. Bae HJ, Park J: Health benefits of improving air quality in the rapidly aging Korean society. Science of the Total Environment 2009, 407:5971-5977.

58. Becares L, Stafford M, Nazroo J: Fear of racism, employment and expected organizational racism: Their association with health. European Journal of Public Health 2009, 19:504-510.

59. Brophy S, Cooksey R, Gravenor MB, Mistry R, Thomas N, Lyons RA, Williams R: Risk factors for childhood obesity at age 5: analysis of the millennium cohort study. BMC public health 2009, 9:467.

60. Corrao G, Zambon A, Parodi A, Mezzanzanica M, Merlino L, Cesana G, Mancia G: Do socioeconomic disparities affect accessing and keeping antihypertensive drug therapy? Evidence from an Italian population-based study. Journal of Human Hypertension 2009, 23:238-244.

61. Kelly Y, Panico L, Bartley M, Marmot M, Nazroo J, Sacker A: Why does birthweight vary among ethnic groups in the UK? Findings from the Millennium Cohort Study. Journal of public health (Oxford, England) 2009, 31:131-137.

62. Kleiser C, Schaffrath Rosario A, Mensink GB, Prinz-Langenohl R, Kurth BM: Potential determinants of obesity among children and adolescents in Germany: results from the cross-sectional KiGGS Study. BMC public health 2009, 9:46.

63. Morgenstern M, Sargent JD, Hanewinkel R: Relation between socioeconomic status and body mass index: Evidence of an indirect path via television use. Archives of Pediatrics and Adolescent Medicine 2009, 163:731-738.

64. Nagel G, Wabitsch M, Galm C, Berg S, Brandstetter S, Fritz M, Klenk J, Peter R, Prokopchuk D, Steiner R, et al: Determinants of obesity in the Ulm Research on Metabolism, Exercise and Lifestyle in Children (URMEL-ICE). European Journal of Pediatrics 2009, 168:1259-1267.

65. Sichieri R, Paula Trotte A, De Souza RA, Veiga GV: School randomised trial on prevention of excessive weight gain by discouraging students from drinking sodas. Public Health Nutrition 2009, 12:197-202.

66. Skelton JA, Cook SR, Auinger P, Klein JD, Barlow SE: Prevalence and Trends of Severe Obesity Among US Children and Adolescents. Academic Pediatrics 2009, 9:322-329.

67. Smith DT, Bartee RT, Dorozynski CM, Carr LJ: Prevalence of overweight and influence of out-of-school seasonal periods on body mass index among American Indian schoolchildren. Preventing chronic disease 2009, 6:A20.

68. Sung SK, Lee SG, Lee KS, Kim DS, Kim KH, Kim KY: First-year treatment adherence among outpatients initiating antihypertensive medication in Korea: Results of a retrospective claims review. Clinical Therapeutics 2009, 31:1309-1320.

69. Voorhees CC, Catellier DJ, Ashwood JS, Cohen DA, Rung A, Lytle L, Conway TL, Dowda M: Neighborhood socioeconomic status and non school physical activity and body mass index in adolescent girls. Journal of Physical Activity and Health 2009, 6:731-740.

70. Wake M, Baur LA, Gerner B, Gibbons K, Gold L, Gunn J, Levickis P, McCallum Z, Naughton G, Sanci L, Ukoumunne OC: Outcomes and costs of primary care surveillance and intervention for overweight or obese children: The LEAP 2 randomised controlled trial. BMJ (Online) 2009, 339:1132.

71. Wong MCS, Jiang JY, Gibbs T, Griffiths SM: Factors associated with antihypertensive drug discontinuation among chinese patients: A cohort study. American Journal of Hypertension 2009, 22:802-810.

72. Wong MCS, Jiang JY, Griffiths SM: Short-term adherence to beta-blocker therapy among ethnic Chinese patients with hypertension: A cohort study. Clinical Therapeutics 2009, 31:2170-2177.

73. Yeaw J, Benner JS, Walt JG, Sian S, Smith DB: Comparing adherence and persistence across 6 chronic medication classes. Journal of Managed Care Pharmacy 2009, 15:728-740.

74. Bourgeois FT, Shannon MW, Valim C, Mandl KD: Adverse drug events in the outpatient setting: An 11-year national analysis. Pharmacoepidemiology and Drug Safety 2010, 19:901-910.

75. Dahl E, Malmberg-Heimonen I: Social inequality and health: The role of social capital. Sociology of Health and Illness 2010, 32:1102-1119.

76. Horney JA, Moore Z, Davis M, MacDonald PDM: Intent to receive pandemic influenza a (H1N1) vaccine, compliance with social distancing and sources of information in NC, 2009. PLoS ONE 2010, 5 (6) (no pagination).

77. Howe LD, Galobardes B, Sattar N, Hingorani AD, Deanfield J, Ness AR, Davey-Smith G, Lawlor DA: Are there socioeconomic inequalities in cardiovascular risk factors in childhood, and are they mediated by adiposity Findings from a prospective cohort study. International Journal of Obesity 2010, 34:1149-1159.

78. Kamate SK, Agrawal A, Chaudhary H, Singh K, Mishra P, Asawa K: Public knowledge, attitude and behavioural changes in an Indian population during the Influenza A (H1N1) outbreak. Journal of Infection in Developing Countries 2010, 4:007-014.

79. Karlsen S, Nazroo JY: Religious and ethnic differences in health: Evidence from the Health Surveys for England 1999 and 2004. Ethnicity and Health 2010, 15:549-568.

80. Lau JTF, Yeung NCY, Choi KC, Cheng MYM, Tsui HY, Griffiths S: Factors in association with acceptability of A/H1N1 vaccination during the influenza A/H1N1 pandemic phase in the Hong Kong general population. Vaccine 2010, 28:4632-4637.

81. Leggat PA, Brown LH, Aitken P, Speare R: Level of concern and precaution taking among australians regarding travel during Pandemic (H1N1) 2009: Results from the 2009 Queensland social survey. Journal of Travel Medicine 2010, 17:291-295.

82. Liao Q, Cowling B, Lam WT, Ng MW, Fielding R: Situational awareness and health protective responses to pandemic influenza A (H1N1) in Hong Kong: A cross-sectional study. PLoS ONE 2010, 5 (10) (no pagination).

83. Schwarzinger M, Flicoteaux R, Cortarenoda S, Obadia Y, Moatti JP: Low acceptability of A/H1N1 pandemic vaccination in french adult population: Did public health policy fuel public dissonance? PLoS ONE 2010, 5 (4) (no pagination).

84. Singh GK, Siahpush M, Kogan MD: Rising Social Inequalities in US Childhood Obesity, 2003-2007. Annals of Epidemiology 2010, 20:40-52.

85. Soskolne V, Manor O: Health inequalities in Israel: Explanatory factors of socio-economic inequalities in self-rated health and limiting longstanding illness. Health and Place 2010, 16:242-251.

86. Stamatakis E, Zaninotto P, Falaschetti E, Mindell J, Head J: Time trends in childhood and adolescent obesity in England from 1995 to 2007 and projections of prevalence to 2015. Journal of Epidemiology and Community Health 2010, 64:167-174.

87. Wong LP, Sam IC: Public sources of information and information needs for pandemic influenza A(H1N1). Journal of community health 2010, 35:676-682.

88. Abu-Zaineh M, Mataria A, Moatti JP, Ventelou B: Measuring and decomposing socioeconomic inequality in healthcare delivery: A microsimulation approach with application to the Palestinian conflict-affected fragile setting. Social Science and Medicine 2011, 72:133-141.

89. Aida J, Kondo K, Kondo N, Watt RG, Sheiham A, Tsakos G: Income inequality, social capital and self-rated health and dental status in older Japanese. Social Science and Medicine 2011, 73:1561-1568.

90. Aldabe B, Anderson R, Lyly-Yrjanainen M, Parent-Thirion A, Vermeylen G, Kelleher CC, Niedhammer I: Contribution of material, occupational, and psychosocial factors in the explanation of social inequalities in health in 28 countries in Europe. Journal of Epidemiology and Community Health 2011, 65:1123-1131.

91. Bjornstrom EES: The neighborhood context of relative position, trust, and self-rated health. Social Science and Medicine 2011, 73:42-49.

92. Bohnert ASB, Valenstein M, Bair MJ, Ganoczy D, McCarthy JF, Ilgen MA, Blow FC: Association between opioid prescribing patterns and opioid overdose-related deaths. JAMA - Journal of the American Medical Association 2011, 305:1315-1321.

93. Chanel O, Luchini S, Massoni S, Vergnaud JC: Impact of information on intentions to vaccinate in a potential epidemic: Swine-origin Influenza A (H1N1). Social Science and Medicine 2011, 72:142-148.

94. Evans CD, Eurich DT, Remillard AJ, Shevchuk YM, Blackburn D: First-Fill Medication Discontinuations and Nonadherence to Antihypertensive Therapy: An Observational Study. American Journal of Hypertension 2011, 03.

95. Ferrante G, Baldissera S, Moghadam PF, Carrozzi G, Trinito MO, Salmaso S: Surveillance of perceptions, knowledge, attitudes and behaviors of the Italian adult population (18-69 years) during the 2009-2010 A/H1N1 influenza pandemic. European Journal of Epidemiology 2011, 26:211-219.

96. Galarce EM, Minsky S, Viswanath K: Socioeconomic status, demographics, beliefs and A(H1N1) vaccine uptake in the United States. Vaccine 2011, 29:5284-5289.

97. Gunasekara FI, Carter K, Blakely T: Change in income and change in self-rated health: Systematic review of studies using repeated measures to control for confounding bias. Social Science and Medicine 2011, 72:193-201.

98. Hallerod B, Gustafsson JE: A longitudinal analysis of the relationship between changes in socio-economic status and changes in health. Social Science and Medicine 2011, 72:116-123.

99. Hamad R, Fernald LC, Karlan DS: Health education for microcredit clients in Peru: a randomized controlled trial. BMC public health 2011, 11:51.

100. Jehu-Appiah C, Aryeetey G, Spaan E, de Hoop T, Agyepong I, Baltussen R: Equity aspects of the National Health Insurance Scheme in Ghana: Who is enrolling, who is not and why? Social Science and Medicine 2011, 72:157-165.

101. Kanadiya MK, Sallar AM: Preventive behaviors, beliefs, and anxieties in relation to the swine flu outbreak among college students aged 18-24 years. Journal of Public Health 2011, 19:139-145.

102. Lin Y, Huang L, Nie S, Liu Z, Yu H, Yan W, Xu Y: Knowledge, Attitudes and Practices (KAP) related to the Pandemic (H1N1) 2009 among Chinese General Population: A Telephone Survey. BMC Infectious Diseases 2011, 11 (no pagination).

103. Meershoek A, Krumeich A, Vos R: The construction of ethnic differences in work incapacity risks: Analysing ordering practices of physicians in the Netherlands. Social Science and Medicine 2011, 72:15-22.

104. Metersky ML, Hunt DR, Kliman R, Wang Y, Curry M, Verzier N, Lyder CH, Moy E: Racial disparities in the frequency of patient safety events: Results from the national medicare patient safety monitoring system. Medical Care 2011, 49:504-510.

105. Myers LB, Goodwin R: Determinants of adults' intention to vaccinate against pandemic swine flu. BMC public health 2011, 11:15.

106. Nemet D, Geva D, Eliakim A: Health promotion intervention in low socioeconomic kindergarten children. Journal of Pediatrics 2011, 158:796-801.e791.

107. O'Connor MJ, Tomlinson M, LeRoux IM, Stewart J, Greco E, Rotheram-Borus MJ: Predictors of alcohol use prior to pregnancy recognition among township women in Cape Town, South Africa. Social Science and Medicine 2011, 72:83-90.

108. Prati G, Pietrantoni L, Zani B: A Social-Cognitive Model of Pandemic Influenza H1N1 Risk Perception and Recommended Behaviors in Italy. Risk Analysis 2011, 31:645-656.

109. Puder JJ, Marques-Vidal P, Schindler C, Zahner L, Niederer I, Burgi F, Ebenegger V, Nydegger A, Kriemler S: Effect of multidimensional lifestyle intervention on fitness and adiposity in predominantly migrant preschool children (Ballabeina): Cluster randomised controlled trial. BMJ (Online) 2011, 343 (7830) (no pagination).

110. Soto Mas F, Olivarez A, Jacobson HE, Hsu CE, Miller J: Risk communication and college students: The 2009 H1N1 pandemic influenza. Preventive Medicine 2011, 52:473-474.

111. Wong MCS, Su X, Jiang JY, Tang JL, Griffiths SM: Profiles of discontinuation and switching of thiazide diuretics: A cohort study among 9398 Chinese hypertensive patients. Hypertension Research 2011, 34:888-893.

112. Becares L, Nazroo J, Albor C, Chandola T, Stafford M: Examining the differential association between self-rated health and area deprivation among white British and ethnic minority people in England. Social Science and Medicine 2012.

113. Cardel M, Willig AL, Dulin-Keita A, Casazza K, Mark Beasley T, Fernandez JR: Parental feeding practices and socioeconomic status are associated with child adiposity in a multi-ethnic sample of children. Appetite 2012, 58:347-353.

114. de Wit MA, Tuinebreijer WC, van Brussel GH, Selten JP: Ethnic differences in risk of acute compulsory admission in Amsterdam, 1996-2005. Social psychiatry and psychiatric epidemiology 2012, 47:111-118.

115. German D, Latkin CA: Social stability and health: exploring multidimensional social disadvantage. Journal of urban health : bulletin of the New York Academy of Medicine 2012, 89:19-35.

116. Herrick H, Thompson H, Kinder J, Madsen KA: Use of SPARK to Promote After-School Physical Activity. Journal of School Health 2012, 82:457-461.

117. Jimenez N, Anderson GD, Shen DD, Nielsen SS, Farin FM, Seidel K, Lynn AM: Is ethnicity associated with morphine's side effects in children? Morphine pharmacokinetics, analgesic response, and side effects in children having tonsillectomy. Paediatric Anaesthesia 2012, 22:669-675.

118. Kumar S, Quinn SC, Kim KH, Musa D, Hilyard KM, Freimuth VS: The social ecological model as a framework for determinants of 2009 H1N1 influenza vaccine uptake in the United States. Health Education and Behavior 2012, 39:229-243.

119. Li M, Chapman GB, Ibuka Y, Meyers LA, Galvani A: Who got vaccinated against H1N1 pandemic influenza? - A longitudinal study in four US cities. Psychology and Health 2012, 27:101-115.

120. Mak KK, Lai CM: Knowledge, risk perceptions, and preventive precautions among Hong Kong students during the 2009 influenza A (H1N1) pandemic. American Journal of Infection Control 2012.

121. Myers LB, Goodwin R: Using a theoretical framework to determine adults' intention to vaccinate against pandemic swine flu in priority groups in the UK. Public Health 2012, 126:S53-S56.

122. Redelings MD, Piron J, Smith LV, Chan A, Heinzerling J, Sanchez KM, Bedair D, Ponce M, Kuo T: Knowledge, attitudes, and beliefs about seasonal influenza and H1N1 vaccinations in a low-income, public health clinic population. Vaccine 2012, 30:454-458.

123. Savoia E, Testa MA, Viswanath K: Predictors of knowledge of H1N1 infection and transmission in the U.S. population. BMC public health 2012, 12:328.

124. Thigpen JL, Yan Q, Liu N, Beasley M, Limdi NA: Racial differences in anticoagulation control and risk of hemorrhage among warfarin users. Pharmacotherapy 2012, 32 (10):e189.

125. Trippe BS, Shepherd MD, Coulter FC, Bhargava A, Brett J, Chu PL, Oyer DS: Efficacy and safety of biphasic insulin aspart 70/30 in type 2 diabetes patients of different race or ethnicity (INITIATEplus trial). Current Medical Research and Opinion 2012, 28:1203-1211.

126. Walter D, Bohmer MM, Reiter S, Krause G, Wichmann O: Risk perception and information-seeking behaviour during the 2009/10 influenza a(H1N1)pdm09 pandemic in Germany. Eurosurveillance 2012, 17:20120329.

127. Williams L, Regagliolo A, Rasmussen S: Predicting psychological responses to influenza A, H1N1 ("swine flu"): the role of illness perceptions. Psychology, health & medicine 2012, 17:383-391.

128. Benoit C, Roth E, Hallgrimsdottir H, Jansson M, Ngugi E, Sharpe K: Benefits and constraints of intimate partnerships for HIV positive sex workers in Kibera, Kenya. International Journal for Equity in Health 2013, 12 (1) (no pagination).

129. Boyd CA, Gazmararian JA, Thompson WW: Knowledge, attitudes, and behaviors of low-income women considered high priority for receiving the novel influenza A (H1N1) vaccine. Maternal and child health journal 2013, 17:852-861.

130. Brooks-Gunn J, Schneider W, Waldfogel J: The great recession and the risk for child maltreatment. Child Abuse and Neglect 2013, 37:721-729.

131. Cengiz E, Xing D, Wong JC, Wolfsdorf JI, Haymond MW, Rewers A, Shanmugham S, Tamborlane WV, Willi SM, Seiple DL, et al: Severe hypoglycemia and diabetic ketoacidosis among youth with type 1 diabetes in the T1D Exchange clinic registry. Pediatric Diabetes 2013, 14:447-454.

132. Dilip TR, Dandona R, Dandona L: The national employment guarantee scheme and inequities in household spending on food and non-food determinants of health in rural India. International Journal for Equity in Health 2013, 12 (1) (no pagination).

133. Lam Y, Broaddus ET, Surkan PJ: Literacy and healthcare-seeking among women with low educational attainment: Analysis of cross-sectional data from the 2011 Nepal demographic and health survey. International Journal for Equity in Health 2013, 12 (1) (no pagination).

134. Richard Kessler E, Shah M, Gruschkus SK, Raju A: Cost and quality implications of opioid-based postsurgical pain control using administrative claims data from a large health system: Opioid-Related adverse events and their impact on clinical and economic outcomes. Pharmacotherapy 2013, 33:383-391.

135. Schwanke Khilji SU, Rudge JW, Drake T, Chavez I, Borin K, Touch S, Coker R: Distribution of selected healthcare resources for influenza pandemic response in Cambodia. International Journal for Equity in Health 2013, 12 (1) (no pagination).

136. Sundmacher L, Kopetsch T: Waiting times in the ambulatory sector - The case of chronically Ill patients. International Journal for Equity in Health 2013, 12 (1) (no pagination).

137. Zere E, Suehiro Y, Arifeen A, Moonesinghe L, Chanda SK, Kirigia JM: Equity in reproductive and maternal health services in Bangladesh. International Journal for Equity in Health 2013, 12 (1) (no pagination).

138. Zhao Y, You J, Wright J, Guthridge SL, Lee AH: Health inequity in the Northern Territory, Australia. International Journal for Equity in Health 2013, 12 (1) (no pagination).

139. Albor C, Uphoff EP, Stafford M, Ballas D, Wilkinson RG, Pickett KE: The effects of socioeconomic incongruity in the neighbourhood on social support, self-esteem and mental health in England. Social Science and Medicine 2014, 111:1-9.

140. Baranowski T, O'Connor T, Johnston C, Hughes S, Moreno J, Chen TA, Meltzer L, Baranowski J: School year versus summer differences in child weight gain: A narrative review. Childhood Obesity 2014, 10:18-24.

141. Branco FLCC, Pereira TM, Delfino BM, Brana AM, Oliart-Guzman H, Mantovani SAS, Martins AC, De Menezes Oliveira CS, Ramalho AA, Codeco CT, Da Silva-Nunes M: Socioeconomic inequalities are still a barrier to full child vaccine coverage in the Brazilian Amazon: A cross-sectional study in Assis Brasil, Acre, Brazil. International Journal for Equity in Health 2014, 13 (1) (no pagination).

142. Cook EJ, Randhawa G, Large S, Guppy A, Chater AM, Pang D: Who uses NHS Direct? Investigating the impact of ethnicity on the uptake of telephone based healthcare. International Journal for Equity in Health 2014, 13 (1) (no pagination).

143. Meghani SH, Kang Y, Chittams J, McMenamin E, Mao JJ, Fudin J: African Americans with cancer pain are more likely to receive an analgesic with toxic metabolite despite clinical risks: A mediation analysis study. Journal of Clinical Oncology 2014, 32:2773-2779.

144. Wang B, Deveaux L, Li X, Marshall S, Chen X, Stanton B: The impact of youth, family, peer and neighborhood risk factors on developmental trajectories of risk involvement from early through middle adolescence. Social Science and Medicine 2014, 106:43-52.

145. Yawson AE, Appiah LK, Yawson AO, Bonsu G, Aluze-Ele S, Owusu Amanhyia NAK, Lartey M, Adjei AA, Lawson AL, Beckwith C, et al: Sex differences in perceived risk and testing experience of HIV in an urban fishing setting in Ghana. International Journal for Equity in Health 2014, 13 (1) (no pagination).

146. Wohland P, Rees P, Nazroo J, Jagger C: Inequalities in healthy life expectancy between ethnic groups in England and Wales in 2001. Ethnicity & health 2015, 20:341-353.

147. Farrants K, Bambra C, Nylen L, Kasim A, Burstrom B, Hunter D: Recommodification, Unemployment, and Health Inequalities: Trends in England and Sweden 1991-2011. International journal of health services : planning, administration, evaluation 2016, 46:300-324.

148. Shamliyan TA, Kane RL: Drug-Related Harms in Hospitalized Medicare Beneficiaries: Results From the Healthcare Cost and Utilization Project, 2000-2008. Journal of patient safety 2016, 12:89-107.

149. Ki M, Lee YH, Kim YS, Shin JY, Lim J, Nazroo J: Socioeconomic inequalities in health in the context of multimorbidity: A Korean panel study. PLoS ONE 2017, 12 (3) (no pagination).

150. Santelli JS, Song X, Garbers S, Sharma V, Viner RM: Global Trends in Adolescent Fertility, 1990-2012, in Relation to National Wealth, Income Inequalities, and Educational Expenditures. Journal of Adolescent Health 2017, 60:161-168.
